# Comparing the prevalence, incidence and severity of mental disorders between deaf and hard-of-hearing and hearing adults aged 18–60: a systematic review

**DOI:** 10.1017/S2045796026100511

**Published:** 2026-03-26

**Authors:** Nino de Ponti, Katharina Diehl, Chris van Klaveren, Ilja Cornelisz, Ralph de Vries, Loes Wauters, Eline Heppe

**Affiliations:** 1 Department of Clinical, Neuro and Developmental Psychology, Amsterdam Public Health Research Institute, Vrije Universiteit Amsterdam, Amsterdam, The Netherlands; 2 Amsterdam Center for Learning Analytics (ACLA), Amsterdam, The Netherlands; 3 Department of Education Sciences, Section Methods and Statistics, Vrije Universiteit Amsterdam, Amsterdam, The Netherlands; 4 Medical Library, Vrije Universiteit Amsterdam, Amsterdam, The Netherlands; 5 Kentalis Academy, Royal Kentalis, Utrecht, The Netherlands; 6 Behavioural Science Institute, Radboud University, Nijmegen, the Netherlands

**Keywords:** deaf and hard-of-hearing, mental disorders, hearing loss, prevalence, systematic review

## Abstract

**Aim:**

Existing reviews on mental health disparities between deaf and hard‐of‐hearing (DHH) and hearing populations have focused predominantly on children, adolescents, or older adults, leaving a gap for working-age adults. We conducted a systematic review comparing the prevalence, incidence, and severity of any DSM-5-TR or ICD-11 mental disorder between DHH and hearing adults aged 18–60 years. We aimed to quantify disparities and examine disorder-specific patterns to inform future research, policy, and service development.

**Methods:**

On 13 December 2025, we searched Ovid Medline, Embase, APA PsycINFO and Web of Science. We included analytical observational studies involving DHH and hearing adults aged 18–60 years, reporting mental disorder prevalence, incidence, or severity. Two researchers independently extracted data, and risk of bias (RoB) was assessed using the modified CLARITY tool. We narratively synthesised findings by aggregating outcomes at the study level using two approaches: summary and majority of the effect directions within a study. Subgroup syntheses examined outcome type, study RoB, age group and mental disorder category.

**Results:**

Sixty studies (*n* = 8 578 466) met inclusion. In the summary-direction synthesis, 58.3% (35/60) of studies reported higher mental disorder outcomes for DHH adults, 21.7% (13/60) found no difference and 20.0% (12/60) had mixed findings; none indicated lower mental disorder outcomes for DHH. Under the majority-direction approach, 65.0% (39/60) showed higher mental disorder outcomes and 35.0% (21/60) no difference. These patterns were consistent across prevalence (62.8–72.1% higher) and severity (61.1% higher). Studies with higher RoB more often reported higher mental disorder outcomes (66.7–72.2%) than lower-RoB studies (54.8–61.9%), though both mirrored the overall synthesis. Effects were similar across younger (61.9–71.4%) and older adult samples (61.1–66.7% higher). Disorder-specific syntheses identified psychotic disorders, post-traumatic stress disorder and suicidal outcomes as having the strongest disparities (≥72.2% higher), followed by general mental disorders, anxiety and depression. Fewer than five studies examined each of the other disorders, thereby limiting conclusions for these disorders.

**Conclusions:**

Most available evidence indicates that the prevalence and severity of mental disorders are higher among DHH adults aged 18–60 years than among hearing adults, with limited evidence on incidence. No studies reported lower aggregated mental disorder outcomes for DHH adults. Addressing these disparities requires targeted intervention research, supported by population-based, longitudinal and (quasi-)experimental studies including comprehensive reporting of participant characteristics. This will inform more tailored interventions, improve screening and ultimately contribute to better mental health and quality of life for DHH adults.

## Introduction

Worldwide, an estimated 1.5 billion deaf and hard-of-hearing (DHH) people live with hearing loss, which ranks among the leading contributors to years lived with disability (GBD 2019 Hearing Loss Collaborators, 2021; GBD 2021 Diseases and Injuries Collaborators, 2024). In this review, we use ‘deaf and hard of hearing (DHH)’ as an inclusive umbrella term referring to people with hearing loss. Capitalised ‘Deaf’ denotes the cultural–linguistic community, whereas lowercase ‘deaf’’ is widely used when referring broadly to all deaf people, particularly when cultural affiliation is unknown (National Association of the Deaf, n.d.). The World Health Organization (WHO) classifies DHH as ranging from mild (20–35 decibels [dB] in the better ear) to profound or total hearing loss (>95 dB in the better ear) (Humes, 2019; World Health Organization, 2021). Of the total DHH population, approximately 1.16 billion (or 77%) have mild hearing loss, while 430 million (or 23%) experience moderate (35–50 dB) or higher levels of hearing loss (World Health Organization, 2021). Across the lifespan, the global prevalence of moderate or higher levels of hearing loss is around 2% for those with early onset (in childhood or adolescence), increases gradually to 2.8% in those aged 40–44 years, and rises more sharply to 8.4% in people aged 55–59 years. Notably, around one-third of DHH people with moderate or higher levels of hearing loss are adults between 18 and 60 years old (GBD 2019 Hearing Loss Collaborators, 2021; Guo *et al.*, 2024).

These DHH adults may face challenges in daily functioning, such as discrimination, accessing information, and stigmatisation, which can increase their vulnerability to mental health problems (de Graaf and Bijl, 2002; Fellinger *et al.*, 2012; Mousley and Chaudoir, 2018). Barriers to effective communication, both early in life and in adulthood, can also contribute to long-term psychosocial and health vulnerabilities among DHH adults, including Deaf signing populations (Kushalnagar *et al.*, 2018, 2020; Rogers *et al.*, 2024). Furthermore, navigating a predominantly hearing world may lead to acculturative stress as DHH adults have to adapt to differing cultural environments (Aldalur *et al.*, 2021; Aldalur and Pick, 2023). Additionally, accessing mental health care remains a significant challenge due to service inaccessibility, lack of cultural sensitivity and limited health literacy (McKee *et al.*, 2015; Smith and Samar, 2016; Morisod *et al.*, 2022).

Given these challenges, several systematic reviews and meta-analyses have examined whether the prevalence, incidence or severity of mental disorders is higher among DHH people than among hearing people. Throughout this review, when referring to two or more of these collectively, we use the umbrella term ‘mental disorder outcomes’. Most of these reviews focused on a single mental disorder and reported higher mental disorder outcomes for depression, anxiety disorders, psychotic disorders and overall psychopathology among DHH children, adolescents and older adults (Theunissen *et al.*, 2014; Linszen *et al.*, 2016; Shoham *et al.*, 2019; Jiang *et al.*, 2020; Lawrence *et al.*, 2020; Trott *et al.*, 2021). While two of these reviews included studies covering the entire lifespan, the majority of the included studies focused on samples of older adults (60 +) or children, yielding limited and only indirect evidence for adults aged 18–60 years (Linszen *et al.*, 2016; Shoham *et al.*, 2019). This highlights a significant gap in the literature regarding the mental health of DHH adults compared to hearing adults. This gap is particularly concerning because employment declines rapidly from age 60, and many adults leave the workforce well before the pension eligibility age (OECD, 2025). Therefore, adults aged 18–60 years represent the majority of the working-age population, for whom mental disorders not only affect individual well-being, functioning and mortality, but also result in significant economic and societal costs, including but not limited to reduced labor market participation and productivity losses (Walker *et al.*, 2015; Christensen *et al.*, 2020; Arias *et al.*, 2022).

Therefore, in the present systematic review, our objective was to compare the prevalence, incidence and severity of mental disorders between DHH and hearing adults aged 18–60 years. We aimed to be the first systematic review to examine the full spectrum of mental disorders as defined by any mental, developmental or behavioural disorder included in the DSM-5-TR or ICD-11 (World Health Organization, 2019; American Psychiatric Association, 2022). By synthesising the overall findings, alongside a disorder-specific breakdown, we aimed to offer a broad evidence base on potential disparities, helping to guide future research, inform policy, support service adaptation, and enable the development of targeted interventions and preventive strategies for DHH adults.

## Methods

The current systematic review was reported according to the PRISMA guidelines, with the checklist provided in Supplement A (Page *et al.*, 2021). The protocol was registered in PROSPERO (CRD42023474624), with four amendments presented in Supplement B.

### Identification and selection of studies

We conducted systematic searches in Ovid Medline, Embase.com, APA PsycInfo (Ebsco) and Web of Science (Core Collection) from inception to 13 December 2025, in collaboration with a medical information specialist (R.d.V.). Search terms (including synonyms and related words) were used as index terms or free-text words: ‘Deaf’, ‘Hard of hearing’, ‘Hearing Disorders’, ‘Persons with Hearing Impairments’, ‘Mental Disorders’, ‘Mental Health’, ‘Prevalence’, ‘Incidence’, ‘Severity’. Publications in all languages were included. Duplicates were removed using Endnote X20.0.1 (Clarivate™), following the Amsterdam Efficient Deduplication (AED) and Bramer methods (Bramer *et al.*, 2016; Otten *et al.*, 2019). The full search strategies are presented in Supplement C. All records were first screened based on title and abstract independently by two researchers (N.d.P. and K.D.) using Rayyan (Ouzzani *et al.*, 2016). Records identified as potentially eligible by either researcher were retrieved for full-text review. Full-text studies were then assessed for inclusion by the same researchers. Disagreements were resolved through discussion, involving a third researcher (C.v.K., I.C., or E.H.) if needed. No language restrictions were applied during the search. During full-text screening, five potentially eligible studies could not be assessed because they were written in a language not understood by the research team. All five were Chinese-language publications.

### Eligibility criteria

We included studies that (a) used a case-control, cohort, or cross-sectional design; (b) examined adult samples with a mean or median age of 18–60 years, at least half the participants within this range, or explicitly described the sample as adults; (c) compared DHH adults with bilateral hearing loss (20–95 dB + in the better ear) with a hearing group. In practice, many primary studies did not report the exact degree of hearing loss, so being DHH could be identified through any method used in the original study; (d) reported differences in the prevalence, incidence, or severity of any mental, behavioural or developmental disorder corresponding to DSM-5-TR or ICD-11 categories, or examined suicidal outcomes such as ideation or attempts (World Health Organization, 2019; American Psychiatric Association, 2022). Clinical diagnosis was not required, and studies were eligible regardless of the type of mental health measure used; no minimum severity threshold was applied. No restrictions on publication date were applied.

Studies were excluded if (a) the DHH group did not meet the age criteria; (b) statistical significance was not reported or effect sizes could not be calculated from the provided data; (c) the comparison group consisted exclusively of people with disabilities (e.g., comparing DHH people only to people with a visual impairment); (d) focused exclusively on neurocognitive disorders; or (e) duplicate use of the same sample for the same outcome to avoid including the same participants across multiple studies reporting on the same outcomes (e.g., the Netherlands Longitudinal Study on Hearing) (Nachtegaal *et al.*, 2009; Stam *et al.*, 2016; Bosdriesz *et al.*, 2018). In such cases, we retained one study per sample and outcome, prioritising longitudinal over cross-sectional designs, clearer age range reporting, larger sample sizes or most outcomes.

### Risk of bias assessment

The potential RoB of the included studies was assessed using a modified version of the CLARITY tool (CLARITY Group at McMaster University, n.d.; van der Ven *et al.*, 2024). This tool is structured across seven domains: (a) sample representativeness of the general population; (b) selection of exposed and non-exposed cohorts; (c) assessment of the exposure; (d) assessment of the outcome; (e) consideration of confounding factors; (f) assessment of confounding factors; and (7) handling missing data. Each domain was rated as either low risk, high risk, or unclear based on the tool’s explanation document (Supplement D). Two raters (N.d.P. and K.D.) independently conducted the assessments, resolving disagreements through discussion.

### Data extraction

Two researchers (N.d.P. and K.D.) independently extracted data, with disagreements resolved through discussion or, if needed, involving a third researcher (C.K., I.C., or E.H.). Extracted data included study characteristics, participant characteristics and empirical findings. Study characteristics included study design (i.e., cross-sectional, case-control, or cohort), publication year, country and continent, recruitment strategy (e.g., schools/universities, register data), sample size per group, examined disorder (e.g., depression, anxiety) and disorder assessment measure (e.g., validated self-report questionnaire, diagnostic interview). Participant characteristics included group descriptions, sample age (range, mean, standard deviation), gender/sex distribution and tinnitus status. For the DHH group, we also extracted the type of audiometric test, preferred language, hearing device usage, pre- or post-lingual onset (as defined in the primary study) and education setting (e.g. mainstream or special education).

Empirical findings included outcome type (i.e., prevalence, symptom severity or incidence), effect direction (e.g., higher prevalence for DHH, no difference), statistical test used (e.g., *t*-test, logistic regression) and reported effect size, or if not reported, data to calculate odds ratios. Prevalence was defined as group differences in the proportion of individuals meeting diagnostic criteria or thresholds; severity as differences in symptom severity questionnaire scores or regression analyses with continuous variables; and incidence as new cases of mental disorders.

### Data synthesis

Odds ratios were calculated for studies without reported effect sizes, using the number of cases in the DHH (exposed) and hearing (control) groups with positive (presence of disorder) and negative (absence of disorder) outcomes (Altman, 1990; MedCalc Software Ltd., n.d.).

We synthesised the results using two complementary approaches, each aggregating findings at the study level so that every study contributed one overall direction of effect. Firstly, the summary-direction approach categorised studies as reporting higher, lower, no difference or mixed mental disorder outcomes for DHH compared to hearing groups, based on statistical significance of the effect direction. Studies were classified as mixed if they reported multiple outcome types, more than one disorder, or subgroup-specific results (e.g., by age or hearing loss degree) with varying directions. Secondly, to address the ambiguity introduced by mixed findings, the majority-direction approach classified studies by the most frequently reported effect direction. When outcomes were evenly split, studies were conservatively reclassified as no difference.

We applied both approaches across all studies (overall effects). Additionally, we conducted separate sensitivity and subgroup syntheses based on study RoB, with lower risk defined as 4–7 low-risk domains (50–100%) and higher risk as 0–3 low-risk domains (0–50%), age groups (younger adults: mean age <40 years or more than 50% of the sample under 40 years; older adults: mean age ≥40 years or more than 50% of the sample over 40 years), and outcome type (prevalence, incidence, and severity). This age distinction was based on global data indicating that hearing loss prevalence increases more sharply after age 40, suggesting that older samples likely include more later-onset hearing loss (GBD 2019 Hearing Loss Collaborators, 2021; Guo *et al.*, 2024). Lastly, we conducted separate syntheses for each mental disorder, again aggregating results at the study level using both synthesis approaches.

## Results

### Selection and inclusion of studies

**Figure 1.**
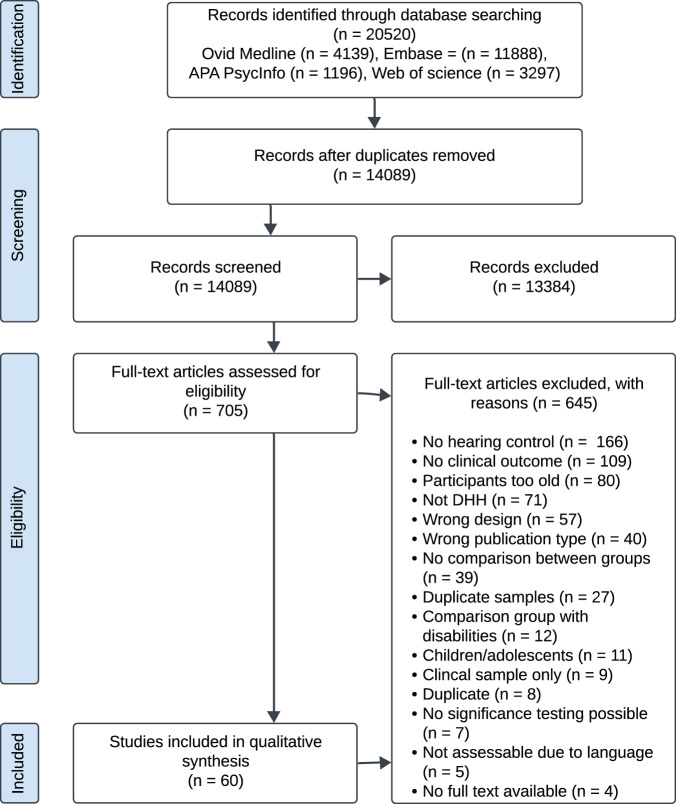
PRISMA flowchart of study identification and selection.

After removing duplicates, we screened a total of 14 089 records, excluding 13 384 based on titles and abstracts. Full texts were retrieved for the remaining 705 records, of which 645 were excluded. The PRISMA flowchart (Fig. 1) details the inclusion process and reasons for full-text exclusions. In total, 60 studies were included for narrative synthesis (full references in Supplement E).

### Study characteristics

Selected characteristics of the 60 included studies are presented in Table 1, and full study-level details for each study are provided in Supplement F. In total, the studies included 8 578 466 participants (median = 3321), of whom 351 707 were DHH adults (median = 269) and 8 226 759 were hearing adults (median = 2489.5). Four studies did not report group-specific numbers. Most studies were cross-sectional (45; 75.0%) and half were published from 2020 onward (30; 50.0%; range: 1989–2025). Geographically, studies were mostly conducted in North America (28; 46.7%) and Europe (19; 31.7%). Terminology used to describe DHH groups varied: 14 studies (23.3%) used terms such as ‘deaf’ or ‘hard of hearing’, while 46 (76.7%) used ‘hearing loss’ or ‘hearing impairment’. Type of audiometric test also varied, with self-report used in 28 studies (46.7%) and audiological assessments such as pure tone audiometry (PTA) in 19 (31.7%). Most studies examined prevalence (40; 66.7%), fewer focused on severity (15; 25.0%) or both (3; 5.0%), and only two (3.3%) focused on incidence. Mental disorder outcomes were typically assessed using validated questionnaires (32; 53.3%). Some characteristics were infrequently reported, limiting further classification: tinnitus status (9; 15.0%), language use (11; 18.3%), hearing device usage (13; 21.7%) and pre- or post-lingual onset (8; 13.3%).

**Table 1.** Selected study characteristics

	Number of studies (percentage)
**Study design**	
Cross sectional	45 (75.0)
Case–control	3 (5.0)
Cohort	12 (20.0)
**Geographical location (continent)**	
North America	28 (46.7)
Europe	19 (31.7)
Asia	9 (15.0)
Other^a^	4 (6.7%)
**Publication year (range 1989–2025)**	
Before 2020	30 (50.0)
After 2020	30 (50.0)
**DHH terminology**	
Terms related to DHH	14 (23.3)
Terms related to Hearing impairment or hearing loss	46 (76.7)
**Type of audiometric test**	
Self-report	28 (46.7)
Audiological assessment (e.g. pure tone audiometry)	19 (31.7)
Diagnostic codes (e.g. insurance registers)	6 (10.0)
No reported instrument	7 (11.7)
**Mental disorder measurement**	
Validated questionnaires	32 (53.3)
Self-report items constructed for the study	10 (16.7)
Diagnostic codes (e.g. ICD − 10 codes in registers)	8 (13.3)
Diagnostic interviews	6 (10.0)
Combination of instruments for different disorders^b^	4 (6.7)
**Outcome types**	
Prevalence	40 (66.7)
Severity	15 (25.0)
Both prevalence and severity	3 (5.0)
Incidence	2 (3.3)

a This included samples from three studies in Russia and Türkiye, and one study conducted in five countries spanning three continents (Africa, Asia and North America).

b Within the same study.

### Risk of bias

The complete RoB assessment for all seven domains is presented in Supplement G. For studies with outcome-specific variation in RoB, the highest risk rating was retained within domains and for the overall assessment. In Domain 1, over half of the studies (31; 51.7%) had high RoB due to voluntary participation, while 28 (46.7%) had low RoB based on representative sources such as national registries. Most studies showed low RoB in Domain 2 (52; 86.7%), as exposed and unexposed groups were generally comparable in source and time frame. In Domain 3, 30 studies (50.0%) had low RoB based on objective or validated hearing loss assessments, while the remaining 30 (50.0%) had high RoB due to self-report measures or insufficient reporting. Most studies (45; 75.0%) had low RoB in Domain 4, using validated questionnaires or registry diagnoses. More than half (35; 58.3%) had low RoB in Domain 5 due to statistical adjustment for confounders. In Domain 6, 40 studies (66.7%) assessed confounding factors with confidence. Over half (36; 60.0%) had unclear RoB in Domain 7, as missing data or response rates were unreported, while 11 studies (18.3%) had low RoB. Overall, 3 studies (5.0%) had low RoB in 0–25% of domains, 15 (25.0%) in 25–50%, 35 (58.3%) in 50–75% and 7 (11.7%) in 75–100%.

### Differences in mental disorder outcomes between DHH and hearing adults

#### Main synthesis

Table 2 presents the results of the summary-direction and majority-direction synthesis approaches, including the overall synthesis and sensitivity syntheses. In the overall effects synthesis using the summary-direction approach, over half of the 60 included studies (35; 58.3%) reported a higher prevalence, incidence, or severity of mental disorders for DHH compared to TH, while the remaining studies found either no difference (13; 21.7%) or mixed findings (12; 20.0%). Similar patterns were found for the majority-direction approach, with increases in both higher for DHH to 65.0% (39), as well as in no differences to 35.0% (21) for the remainder of studies. Therefore, mixed findings consisted of higher for DHH and no difference findings. Notably, not a single study in either synthesis approach reported lower aggregated mental disorder outcomes for DHH groups.

**Table 2.** Differences in mental disorder outcomes between DHH and hearing adults

Synthesis	*N*	Higher for DHH % (*N*)	Lower for DHH % (*N*)	No difference % (*N*)	Mixed % (N)
***Summary-direction approach***					
Overall effects	60	58.3% (35)	0.0% (0)	21.7% (13)	20.0% (12)
Higher RoB	18	66.7% (12)	0.0% (0)	22.2% (4)	11.1% (2)
Lower RoB	42	54.8% (23)	0.0% (0)	21.4% (9)	23.8% (10)
Younger adults (mean age < 40)	21	61.9% (13)	0.0% (0)	19.0% (4)	19.0% (4)
Older adults (mean age ≥ 40)	36	61.1% (22)	0.0% (0)	25.0% (9)	13.9% (5)
Only prevalence	43	62.8% (27)	0.0% (0)	11.6% (5)	25.6% (11)
Only incidence	2	0.0% (0)	0.0% (0)	50.0% (1)	50.0% (1)
Only severity	18	61.1% (11)	0.0% (0)	38.9% (7)	0.0% (0)
**Majority-direction approach**					
Overall effects	60	65.0% (39)	0.0% (0)	35.0% (21)	-
Higher RoB	18	72.2% (13)	0.0% (0)	27.8% (5)	-
Lower RoB	42	61.9% (26)	0.0% (0)	38.1% (16)	-
Younger adults (mean age < 40)	21	71.4% (15)	0.0% (0)	28.6% (6)	-
Older adults (mean age ≥ 40)	36	66.7% (24)	0.0% (0)	33.3% (12)	-
Only prevalence	43	72.1% (31)	0.0% (0)	27.9% (12)	-
Only incidence	2	0.0% (0)	0.0% (0)	100% (2)	-
Only severity	18	61.1% (11)	0.0% (0)	38.9% (7)	-

Note: Results are aggregated at the study level; *N* = Number of studies; RoB = Risk of Bias; Lower age = mean age lower than 40 years or more than 50% of sample under 40; Higher age = mean age higher than 40 years or more than 50% of sample over 40). Studies with equal findings were regarded as no difference per outcome in the majority-direction synthesis: overall effects (*N* = 2), only prevalence (*N* = 1), Only incidence (*N* = 1), Lower RoB (*N* = 2), Younger adults (*N* = 1) Older adults (*N* = 1); Three studies did not report data for age group classification, all reported mixed results (majority-direction: all three no difference).

In the sensitivity analysis by RoB, studies with higher RoB more frequently reported higher mental disorder outcomes for DHH (summary: 66.7%—majority: 72.2%) than lower RoB studies (54.8%—61.9%). Mixed findings were more frequent among lower RoB studies (23.8%) than higher RoB studies (11.1%), reflecting higher and no difference mental disorder outcomes with two ties regarded as no difference. In the subgroup analysis by age, over half of studies in both younger (mean age < 40) and older (mean age ≥ 40) adult samples showed higher mental disorder outcomes for DHH, with majority-direction results of 71.4% (younger adults) and 66.7% (older adults), while the remaining studies showed no difference. Lastly, in the sensitivity syntheses by outcome type, we observed similar patterns for prevalence and severity as the overall synthesis. Most studies reported higher prevalence (62.8%—72.1%) and severity (61.1%) of mental disorders for DHH in both syntheses approaches. For prevalence, 25.6% of studies showed mixed findings; these reflected combinations of higher prevalence for DHH and no difference. For severity, findings were identical across approaches due to the absence of mixed results. Incidence was reported in only two studies, one showing no difference and one mixed (treated as no difference in majority-direction synthesis). Consistent with the overall effects, across all sensitivity and subgroup syntheses, no study reported lower aggregated mental disorder outcomes for DHH groups compared to hearing groups.

#### Disorder-specific synthesis

Table 3 presents the disorder-specific results of both synthesis approaches. Six mental disorders were examined in at least five studies: depression (33 studies), anxiety (15 studies), psychotic disorders (11 studies), suicidal outcomes (8 studies), general mental disorders (7 studies), and post-traumatic stress disorder (PTSD; 5 studies). In the majority-direction synthesis, all mixed findings from the summary-direction approach were reclassified as either higher for DHH or no difference (including ties). Across disorders, PTSD (80.0%), suicidal outcomes (75.0%), and psychotic disorders (72.2%—81.8%) showed the greatest tendency towards higher mental disorder outcomes for DHH. Moreover, for anxiety (53.3%) and general mental disorders (57.1%), more than half of the studies indicated higher mental disorder outcomes for DHH. Lastly, for depression, just under half of the studies (48.5%) reported higher mental disorder outcomes for DHH, while the remaining studies indicated no difference. Across all six disorders, none showed lower aggregated mental disorder outcomes for DHH groups.

**Table 3.** Disorder-specific synthesis

Mental disorder	Synthesis approach	N	Higher for DHH % (*N*)	Lower for DHH % (*N*)	No difference % (*N*)	Mixed % (*N*)
Depression	Summary	33	48.5% (16)	0.0% (0)	33.3% (11)	18.2% (6)
	Majority	33	48.5% (16)	0.0% (0)	51.5% (17)	-
Anxiety	Summary	15	53.3% (8)	0.0% (0)	40.0% (6)	6.7% (1)
	Majority	15	53.3% (8)	0.0% (0)	46.7% (7)	-
Psychotic disorders	Summary	11	72.2% (8)	0.0% (0)	9.1% (1)	18.2% (2)
	Majority	11	81.8% (9)	0.0% (0)	18.2% (2)	-
Suicidal outcomes	Summary	8	75.0% (6)	0.0% (0)	12.5% (1)	12.5% (1)
	Majority	8	75.0% (6)	0.0% (0)	25.0% (2)	-
General mental disorders	Summary	7	57.1% (4)	0.0% (0)	28.6% (2)	14.3% (1)
	Majority	7	57.1% (4)	0.0% (0)	42.9% (3)	-
Post-traumatic stress disorder	Summary	5	80.0% (4)	0.0% (0)	0.0% (0)	20.0% (1)
	Majority	5	80.0% (4)	0.0% (0)	20.0% (1)	-
Substance use disorders	Summary	4	50.0% (2)	0.0% (0)	0.0% (0)	50.0% (2)
	Majority	4	75.0% (3)	0.0% (0)	25.0% (1)	-
Sleep-wake disorders	Summary	4	50.0% (2)	0.0% (0)	25.0% (1)	25.0% (1)
	Majority	4	50.0% (2)	0.0% (0)	50.0% (2)	-
Somatic Symptom disorder	Identical	4	50.0% (2)	0.0% (0)	50.0% (2)	-
Depression and anxiety pooled	Identical	3	66.7% (2)	0.0% (0)	33.3% (1)	-
Sexual dysfunctions	Identical	2	50.0% (1)	0.0% (0)	50.0% (1)	-
Intellectual developmental disorder	Identical	1	100.0% (1)	0.0% (0)	0.0% (0)	-
Obsessive compulsive disorder	Identical	1	0.0% (0)	0.0% (0)	100.0% (1)	-

Note: Results are aggregated at the study level; *N* = number of studies; *Identical* = Summary and Majority effect directions were the same (i.e., no mixed findings were present within the study); Equal findings studies regarded as no difference per outcome in the majority-direction synthesis: Depression (*N* = 2), General mental disorders (*N* = 1), Post traumatic stress disorder (*N* = 1), Substance use disorders (*N* = 1).

Other disorders were examined in fewer than five studies each, including somatic symptom disorder, substance use disorders, and sleep–wake disorders (4 studies each), depression and anxiety pooled (3 studies), sexual dysfunctions (2 studies), and intellectual developmental disorder and obsessive compulsive disorder (1 each). For most disorders, the predominant effect was higher mental disorder outcomes for DHH, followed by no difference. Consistent with all earlier syntheses, no disorder-specific synthesis showed lower aggregated mental disorder outcomes for DHH.

## Discussion

In this systematic review covering 60 studies, we aimed to synthesise the existing evidence for differences in mental disorder prevalence, incidence, or severity between DHH and hearing adults aged 18 to 60. Most studies (58.3%–65.0%) reported higher mental disorder outcomes for DHH adults, while the remaining studies primarily reported no differences. Notably, not a single study showed lower mental disorder outcomes for DHH in any synthesis approach. These patterns were consistent across prevalence and severity outcomes. Only two studies examined incidence, both finding no group differences in the majority-direction synthesis. In studies with lower risk of bias, around 60% reported higher mental disorder outcomes for DHH adults, compared to around 70% in higher-risk studies; yet both patterns broadly aligned with the overall synthesis. Results were also consistent across age groups, with approximately 60%–70% of studies reporting higher mental disorder outcomes for both younger and older DHH adults. Disorder-specific syntheses aligned with the overall synthesis, with no aggregated effect direction resulting in lower mental disorder outcomes for DHH. For the more commonly studied disorders, depression, anxiety, psychotic disorders, suicidal outcomes, general mental disorders, and post-traumatic stress disorder, the proportion of studies reporting higher mental disorder outcomes for DHH adults ranged from 48.5% for depression to 72.2%–81.8% for psychotic disorders. Other disorders, such as sleep–wake disorders and substance use disorders, were examined in fewer than five studies each, limiting the reliability of conclusions for these categories.

These findings align with previous systematic research on depression, anxiety, psychotic disorders, and general psychopathology, which also indicates a potential higher prevalence or severity of mental disorders for DHH people compared to hearing people, particularly for children, adolescents, and older adults (Theunissen *et al.*, 2014; Linszen *et al.*, 2016; Shoham *et al.*, 2019; Jiang *et al.*, 2020; Lawrence *et al.*, 2020; Trott *et al.*, 2021). Our findings indicate that elevated mental health risks are not confined to early developmental stages, but may arise or persist throughout adulthood. A separate question concerns the influence of age of onset of hearing loss. Few studies reported this information, limiting firm conclusions about its potential impact on mental health (de Graaf and Bijl, 2002; Ciesla *et al.*, 2016; Linszen *et al.*, 2016). Still, our comparison of younger and older adult samples suggested broadly similar patterns in mental disorder outcomes. Given that the prevalence of hearing loss remains relatively stable before age 40, it is plausible that a sizeable portion of younger adults in these samples experienced earlier-onset hearing loss, which provides suggestive evidence that mental disorder outcomes are broadly similar for people with both early and later onset hearing loss (GBD 2019 Hearing Loss Collaborators, 2021; World Health Organization, 2021). However, this interpretation remains speculative and should be approached with caution.

This systematic review had several strengths. First, it addressed a key gap in the literature by focusing specifically on adults aged 18 to 60, a substantial proportion of the working-age population whose mental health is critical not only for individual well-being but also for broader societal and economic functioning (Walker *et al.*, 2015; Christensen *et al.*, 2020; Arias *et al.*, 2022). Second, it is the first to synthesise mental disorder outcomes across the full diagnostic spectrum in adults. By including studies on prevalence, incidence, and severity for a wide range of disorders, we provide a comprehensive overview of current evidence on mental health disparities between DHH and hearing adults. Third, applying two complementary synthesis approaches reduced ambiguity from within-study variation. Finally, Subgroup analyses by outcome type, risk of bias, and age enabled a more nuanced understanding of how patterns vary across methodological approaches and subgroups.

This systematic review also has some limitations. First, there was considerable heterogeneity between studies, including differences in study design, exposure and outcome measurement, and control for confounding. Many studies relied on non-validated, single-item self-report measures of hearing loss, often lacking detail on severity or laterality, possibly leading to misclassification (Tremblay *et al*. 2015; Musiek *et al*. 2017; World Health Organization, 2021). Second, over three quarter of the studies were conducted in North America and Europe, potentially limiting generalisability to global DHH populations. Third, age of onset of hearing loss was rarely reported, restricting direct examination of whether early or late onset DHH may explain differences in mental disorder outcomes. Fourth, a proportion of studies reported no statistically significant group differences, yet many of these studies had small sample sizes or many subgroup analyses, thereby increasing the risk of Type II error. These findings should therefore be interpreted with caution, as the absence of detected differences may reflect limited statistical power rather than a true absence of disparities.

Given the findings, several recommendations can be made for future research, policy, and practice. First, differences in mental disorder outcomes between DHH and hearing people remain unknown for many disorders. For example, differences in eating disorders and personality disorders have not been studied, and several others (e.g., OCD, sleep-wake disorders) have only been minimally explored. These gaps need to be filled, as understanding the full scope of mental health disparities across all mental disorders is crucial to ensuring comprehensive care and targeted prevention efforts for the DHH population. Secondly, future research could benefit from employing longitudinal and population-based designs to better track mental health trajectories over time. Additionally, quasi-experimental approaches, such as matching studies, can help improve causal inference by accounting for confounding factors. Thirdly, sample diversity and representativeness should be prioritised, as key DHH characteristics (e.g., degree of hearing loss, language preference, age of onset, type of education) are rarely reported, limiting the generalisability of findings. Finally, there is an urgent need for mental health intervention research. Only a few RCTs have been conducted to date, primarily in children or older adults, leaving working-age DHH adults underrepresented (Trott *et al.*, 2025). Future interventions, such as psychotherapy, should be accessible, culturally and linguistically appropriate, and developed in collaboration with the DHH community (Fellinger *et al.*, 2012; James *et al.*, 2022a).

In terms of policy and practice, our findings highlight the importance of ensuring adequate and equitable access to mental health care for DHH adults, who may face more elevated risks for mental disorders. Yet, significant barriers remain, including communication challenges, cultural mismatches, and stigma, which often hinder access to appropriate care (Kuenburg *et al.*, 2016; Mousley and Chaudoir, 2018; James *et al.*, 2022b). To address these issues, mental health services ought to be adapted to better meet the needs of DHH adults through more accessible, culturally sensitive, and linguistically appropriate approaches (Sheppard, 2014; Kuenburg *et al.*, 2016; James *et al.*, 2022b). In addition, routine screening for symptoms of mental disorders should be implemented during healthcare encounters, including in settings where hearing loss is assessed, to ensure not only identification of those with elevated symptoms, but also early identification of those at elevated risk (Sheppard, 2014; Dethmers *et al.*, 2025). But, without integrated and inclusive support, the mental health needs of DHH adults will continue to go unmet (World Health Organization, 2021). Policies should therefore prioritise investment in tailored, evidence-informed services that are co-designed with DHH communities.

To conclude, this review shows that the majority of the current evidence indicates higher prevalence or severity of mental disorders for DHH adults aged 18–60 years compared to hearing adults, while evidence on incidence remains too limited to draw firm conclusions. Notably, none of the aggregated findings suggested lower mental disorder outcomes for DHH adults. Addressing these persistent disparities will require intervention research, along with more population-based, longitudinal and quasi-experimental designs, incorporating comprehensive reporting of participant characteristics. This would substantially advance understanding, support the development of tailored interventions, and ultimately contribute to improved mental health and quality of life for DHH adults.

## Supporting information

10.1017/S2045796026100511.sm001 de Ponti et al. supplementary material de Ponti et al. supplementary material

## Data Availability

All data and information supporting this study are presented in the main text, tables and supplementary materials. Additional information can be requested from the corresponding author (n.de.ponti@vu.nl).

## Supplementary material

The supplementary material for this article can be found at https://doi.org/10.1017/S2045796026100511
