# Supplementary material for: Comparing the prevalence, incidence and severity of mental disorders between deaf and hard-of-hearing and hearing adults aged 18–60: a systematic review
Source: Epidemiol Psychiatr Sci. 2026 Mar 26;35:e15. doi: 10.1017/S2045796026100511 (PMC13122551; doi:10.1017/S2045796026100511)
Supplement: de Ponti et al. supplementary material [file S2045796026100511sup001.pdf]

Supplement A

PRISMA checklist

Section and Topic	Item #	Checklist item	Location where item is reported
**TITLE**			
Title	1	Identify the report as a systematic review.	Title
**ABSTRACT**			
Abstract	2	See the PRISMA 2020 for Abstracts checklist.	
**INTRODUCTION**			
Rationale	3	Describe the rationale for the review in the context of existing knowledge.	Introduction (second to last paragraph)
Objectives	4	Provide an explicit statement of the objective(s) or question(s) the review addresses.	Introduction (last paragraph)
**METHODS**			
Eligibility criteria	5	Specify the inclusion and exclusion criteria for the review and how studies were grouped for the syntheses.	Methods (eligibility criteria)
Information sources	6	Specify all databases, registers, websites, organisations, reference lists and other sources searched or consulted to identify studies. Specify the date when each source was last searched or consulted.	Methods (first paragraph)
Search strategy	7	Present the full search strategies for all databases, registers and websites, including any filters and limits used.	Supplement C
Selection process	8	Specify the methods used to decide whether a study met the inclusion criteria of the review, including how many reviewers screened each record and each report retrieved, whether they worked independently, and if applicable, details of automation tools used in the process.	Methods (Identification and selection of studies)
Data collection process	9	Specify the methods used to collect data from reports, including how many reviewers collected data from each report, whether they worked independently, any processes for obtaining or confirming data from study investigators, and if applicable, details of automation tools used in the process.	Methods (Data extraction)
Data items	10a	List and define all outcomes for which data were sought. Specify whether all results that were compatible with each outcome domain in each study were sought (e.g. for all measures, time points, analyses), and if not, the methods used to decide which results to collect.	Methods (Data extraction)
	10b	List and define all other variables for which data were sought (e.g. participant and intervention characteristics, funding sources). Describe any assumptions made about any missing or unclear information.	Methods (Data extraction)
Study risk of bias	11	Specify the methods used to assess risk of bias in the included studies, including details of the tool(s) used, how many reviewers assessed	Methods (Risk

Section and Topic	Item #	Checklist item	Location where item is reported
assessment		each study and whether they worked independently, and if applicable, details of automation tools used in the process.	of bias assessment)
Effect measures	12	Specify for each outcome the effect measure(s) (e.g. risk ratio, mean difference) used in the synthesis or presentation of results.	Methods (Data synthesis)
Synthesis methods	13a	Describe the processes used to decide which studies were eligible for each synthesis (e.g. tabulating the study intervention characteristics and comparing against the planned groups for each synthesis (item #5)).	Methods (Data synthesis)
	13b	Describe any methods required to prepare the data for presentation or synthesis, such as handling of missing summary statistics, or data conversions.	Methods (Data synthesis)
	13c	Describe any methods used to tabulate or visually display results of individual studies and syntheses.	Methods (Data synthesis)
	13d	Describe any methods used to synthesize results and provide a rationale for the choice(s). If meta-analysis was performed, describe the model(s), method(s) to identify the presence and extent of statistical heterogeneity, and software package(s) used.	Methods (Data synthesis)
	13e	Describe any methods used to explore possible causes of heterogeneity among study results (e.g. subgroup analysis, meta-regression).	Methods (Data synthesis)
	13f	Describe any sensitivity analyses conducted to assess robustness of the synthesized results.	Methods (Data synthesis)
Reporting bias assessment	14	Describe any methods used to assess risk of bias due to missing results in a synthesis (arising from reporting biases).	NA
Certainty assessment	15	Describe any methods used to assess certainty (or confidence) in the body of evidence for an outcome.	NA
**RESULTS**			
Study selection	16a	Describe the results of the search and selection process, from the number of records identified in the search to the number of studies included in the review, ideally using a flow diagram.	Results (Selection and inclusion of studies) & Figure 1
	16b	Cite studies that might appear to meet the inclusion criteria, but which were excluded, and explain why they were excluded.	NA
Study characteristics	17	Cite each included study and present its characteristics.	Supplement E & Supplement F
Risk of bias in studies	18	Present assessments of risk of bias for each included study.	Supplement G
Results of individual studies	19	For all outcomes, present, for each study: (a) summary statistics for each group (where appropriate) and (b) an effect estimate and its precision (e.g. confidence/credible interval), ideally using structured tables or plots.	Results (Differences in mental disorder

Section and Topic	Item #	Checklist item	Location where item is reported
			outcomes between DHH and hearing adults), Table 2 and Table 3
Results of syntheses	20a	For each synthesis, briefly summarise the characteristics and risk of bias among contributing studies.	NA
	20b	Present results of all statistical syntheses conducted. If meta-analysis was done, present for each the summary estimate and its precision (e.g. confidence/credible interval) and measures of statistical heterogeneity. If comparing groups, describe the direction of the effect.	NA
	20c	Present results of all investigations of possible causes of heterogeneity among study results.	NA
	20d	Present results of all sensitivity analyses conducted to assess the robustness of the synthesized results.	Results (Differences in mental disorder outcomes between DHH and hearing adults), Table 2 and Table 3
Reporting biases	21	Present assessments of risk of bias due to missing results (arising from reporting biases) for each synthesis assessed.	NA
Certainty of evidence	22	Present assessments of certainty (or confidence) in the body of evidence for each outcome assessed.	NA
**DISCUSSION**			
Discussion	23a	Provide a general interpretation of the results in the context of other evidence.	Discussion (first two paragraphs)
	23b	Discuss any limitations of the evidence included in the review.	Discussion (fourth paragraph)
	23c	Discuss any limitations of the review processes used.	NA
	23d	Discuss implications of the results for practice, policy, and future research.	Discussion (fifth paragraph)
**OTHER INFORMATION**			
Registration and protocol	24a	Provide registration information for the review, including register name and registration number, or state that the review was not registered.	Methods (first paragraph)

Section and Topic	Item #	Checklist item	Location where item is reported
	24b	Indicate where the review protocol can be accessed, or state that a protocol was not prepared.	NA
	24c	Describe and explain any amendments to information provided at registration or in the protocol.	Methods (first paragraph) & Supplement B
Support	25	Describe sources of financial or non-financial support for the review, and the role of the funders or sponsors in the review.	Financial Support
Competing interests	26	Declare any competing interests of review authors.	Competing interests
Availability of data, code and other materials	27	Report which of the following are publicly available and where they can be found: template data collection forms; data extracted from included studies; data used for all analyses; analytic code; any other materials used in the review.	Availability of data and materials

From: Page MJ, McKenzie JE, Bossuyt PM, Boutron I, Hoffmann TC, Mulrow CD, et al. The PRISMA 2020 statement: an updated guideline for reporting systematic reviews. BMJ 2021;372:n71. doi: 10.1136/bmj.n71. This work is licensed under CC BY 4.0. To view a copy of this license, visit https://creativecommons.org/licenses/by/4.0/

Supplement B

Amendments from the protocol

First, in the original protocol, we planned to assess the risk of bias in the included studies using the Newcastle-Ottawa Scale. However, upon recognizing that most of the studies were cross-sectional, we opted to use the CLARITY tool, which is better suited for evaluating the risk of bias in studies with various research designs.

Second, the protocol initially included working-age adults aged 18 to 67 years. We revised this range to 18 to 60 years for two reasons. First, studies with mean ages above 60 frequently included older adults with age-related hearing loss, a population for which mental health differences between DHH and hearing adults have already been systematically examined. Second, OECD data indicate that although employment among adults aged 55 and over has increased, labour market participation declines rapidly from age 60, with many individuals leaving the workforce well before reaching pension eligibility (OECD, 2025). Given these considerations, we set the upper age limit at 60.

Third, although not explicitly specified in the protocol, we excluded studies focusing exclusively on neurocognitive disorders, which were outside the intended scope of this review given its focus on a primarily working-age adult population.

Fourth, the original protocol did not specify any language restrictions. During full-text screening, five potentially eligible studies were identified that could not be assessed because they were written in Chinese, a language not understood by the authors.

Reference

OECD (2025) OECD Employment Outlook 2025: Can We Get Through the Demographic Crunch? Paris. Available at https://doi.org/10.1787/194a947b-en (accessed 25 November 2025).

Supplement C

Searches

Ovid Medline Search Results – 13 December 2025

Search	Query	Results
#5	**4 not ((exp Adolescent/ or exp Child/ or exp Infant/ or (adolescen* or child* or schoolchild* or infant* or girl* or boy or boys or teen or teens or teenager* or youth* or pediatr* or paediatr* or puber*).ab,ti,kf.) not (exp Adult/ or (adult* or man or men or woman or women).ab,ti,kf.))**	4,139
#4	**1 and 2 and 3**	5,522
#3	**exp "Severity of Illness Index"/ or exp "Regression Analysis"/ or Morbidity/ or exp Prevalence/ or exp Incidence or ((rate* or validat* or diagnos*) adj3 (tool* or assess* or measur* or instrument* or survey* or questionnaire* or index or standard* or analys* or criteri*)).ab,ti,kf. or ("logistic regress*" or "regression analys*" or sampl* or prevalen* or inciden* or severit*).ab,ti,kf.**	6,719,081
#2	**exp "Mental Health"/ or exp "Mental Disorders"/ or exp "Anxiety"/ or exp "Self-Injurious Behavior"/ or exp "Depression"/ or exp "Psychopathology"/ or mental.ab,ti,kf. or psychiatric.ab,ti,kf. or depress*.ab,ti,kf. or anxiet*.ab,ti,kf. or "suicid*".ab,ti,kf. or agoraphobia.ab,ti,kf. or phobia.ab,ti,kf. or "obsessive compuls*".ab,ti,kf. or "anorexia nervos*".ab,ti,kf. or "bulimia nervos*".ab,ti,kf. or "binge-eat*".ab,ti,kf. or "eating disorder*".ab,ti,kf. or "bipolar disorder*".ab,ti,kf. or "manic disorder*".ab,ti,kf. or "personality disorder*".ab,ti,kf. or "psychotic disorder*".ab,ti,kf. or psychos*.ab,ti,kf. or schizo*.ab,ti,kf. or "sleep disorder*".ab,ti,kf. or "substance-related disorder*".ab,ti,kf. or "stress disorder*".ab,ti,kf. or "attention deficit".ab,ti,kf. or "disruptive behav*".ab,ti,kf. or "conduct disorder*".ab,ti,kf. or "mood disorder*".ab,ti,kf. or autism.ab,ti,kf. or asperger*.ab,ti,kf.**	2,742,188
#1	**exp "Hearing disorders"/ or exp "Persons With Hearing Impairments"/ or ((exp "Hearing"/) and (impair*.ab,ti,kf. or disorder*.ab,ti,kf. or disabled.ab,ti,kf. or loss*.ab,ti,kf. or defect*.ab,ti,kf. or damage.ab,ti,kf. or handicap*.ab,ti,kf. or function*.ab,ti,kf. or dysfunction*.ab,ti,kf.)) or ((hearing.ab,ti,kf.) adj3 (impair*.ab,ti,kf. or disorder*.ab,ti,kf. or disabled.ab,ti,kf. or loss*.ab,ti,kf. or defect*.ab,ti,kf. or damage.ab,ti,kf. or handicap*.ab,ti,kf. or function*.ab,ti,kf. or dysfunction*.ab,ti,kf.)) or "hard-of-hearing".ab,ti,kf. or deaf*.ab,ti,kf. or "d deaf*".ab,ti,kf. or "auditory rehab*".ab,ti,kf. or "auditory disab*".ab,ti,kf. or "auditory impair*".ab,ti,kf. or "auditory handicap*".ab,ti,kf. or "hypoacus*".ab,ti,kf. or exp "Hearing Aids"/ or exp "Correction of Hearing Impairment"/ or "hearing aid*".ab,ti,kf. or "hearing implant*".ab,ti,kf. or "hearing prosthes*".ab,ti,kf. or "hearing device*".ab,ti,kf. or "cochlear implant*".ab,ti,kf. or "cochlear**	170,346

Search	Query	Results
	prosthes*".ab,ti,kf. or "cochlear device*".ab,ti,kf. or ((auditory.ab,ti,kf.) and (implant*.ab,ti,kf. or prosthes*.ab,ti,kf.))	

Embase.com Search Results – 13 December 2025

Search	Query	Results
#6	#5 NOT ('conference abstract'/it OR 'conference review'/it) NOT 'clinical trial':dtype	11,888
#5	#4 NOT (('adolescent'/exp OR 'child'/exp OR (adolescen* OR child* OR schoolchild* OR infant* OR girl* OR boy OR boys OR teen OR teens OR teenager* OR youth* OR pediatr* OR paediatr* OR puber*):ab,ti,kw) NOT ('adult'/exp OR (adult* OR man OR men OR woman OR women):ab,ti,kw))	15,475
#4	#1 AND #2 AND #3	19,332
#3	'severity of illness index'/exp OR 'regression analysis'/exp OR 'morbidity'/de OR 'prevalence'/de OR 'incidence'/de OR ((rate* OR validat* OR diagnos*) NEAR/3 (tool* OR assess* OR measur* OR instrument* OR survey* OR questionnaire* OR index OR standard* OR analys* OR criteri*)):ab,ti,kw OR ("logistic regress*" or "regression analys*" or sampl* or prevalen* or inciden* or severit*):ab,ti,kw	9,549,345
#2	'mental health'/exp OR 'mental disease'/exp OR 'anxiety'/exp OR 'automutilation'/exp OR 'suicidal behavior'/exp OR mental:ab,ti,kw OR psychiatric:ab,ti,kw OR depress*:ab,ti,kw OR anxiet*:ab,ti,kw OR "suicid*":ab,ti,kw OR agoraphobia:ab,ti,kw OR phobia:ab,ti,kw OR "obsessive compuls*":ab,ti,kw OR "anorexia nervos*":ab,ti,kw OR "bulimia nervos*":ab,ti,kw OR "binge-eat*":ab,ti,kw OR "eating disorder*":ab,ti,kw OR "bipolar disorder*":ab,ti,kw OR "manic disorder*":ab,ti,kw OR "personality disorder*":ab,ti,kw OR "psychotic disorder*":ab,ti,kw OR psychos*:ab,ti,kw OR schizo*:ab,ti,kw OR "sleep disorder*":ab,ti,kw OR "substance-related disorder*":ab,ti,kw OR "stress disorder*":ab,ti,kw OR "attention deficit":ab,ti,kw OR "disruptive behav*":ab,ti,kw OR "conduct disorder*":ab,ti,kw OR "mood disorder*":ab,ti,kw OR autism:ab,ti,kw OR asperger*:ab,ti,kw	4,393,608
#1	'hearing disorder'/exp OR 'hearing impaired person'/exp OR ('hearing'/exp AND (impair*:ab,ti,kw OR disorder*:ab,ti,kw OR disabled:ab,ti,kw OR loss*:ab,ti,kw OR defect*:ab,ti,kw OR damage:ab,ti,kw OR handicap*:ab,ti,kw OR function*:ab,ti,kw OR dysfunction*:ab,ti,kw)) OR ((hearing) NEAR/3 (impair* OR disorder* OR disabled OR loss* OR defect* OR damage OR handicap* OR function* OR dysfunction*)):ab,ti,kw OR "hard-of-hearing":ab,ti,kw OR deaf*:ab,ti,kw OR "d deaf*":ab,ti,kw OR "auditory rehab*":ab,ti,kw OR "auditory disab*":ab,ti,kw OR "auditory impair*":ab,ti,kw OR "auditory handicap*":ab,ti,kw OR	283,181

Search	Query	Results
	"hypoacus*":ab,ti,kw OR 'hearing aid'/exp OR 'auditory rehabilitation'/exp OR 'auditory implant'/exp OR "hearing aid*":ab,ti,kw OR "hearing implant*":ab,ti,kw OR "hearing prosthes*":ab,ti,kw OR "hearing device*":ab,ti,kw OR "cochlear implant*":ab,ti,kw OR "cochlear prosthes*":ab,ti,kw OR "cochlear device*":ab,ti,kw OR (auditory:ab,ti,kw AND (implant*:ab,ti,kw OR prosthes*:ab,ti,kw))	

APA PsycInfo (Ebsco) Search Results – 13 December 2025

Search	Query	Results
S5	S4 NOT ((TI ((adolescen* OR child* OR schoolchild* OR infant* OR girl* OR boy OR boys OR teen OR teens OR teenager* OR youth* OR pediatr* OR paediatr* OR puber*) NOT (adult* OR man OR men OR woman OR women))) OR (AB ((adolescen* OR child* OR schoolchild* OR infant* OR girl* OR boy OR boys OR teen OR teens OR teenager* OR youth* OR pediatr* OR paediatr* OR puber*) NOT (adult* OR man OR men OR woman OR women))) OR (KW ((adolescen* OR child* OR schoolchild* OR infant* OR girl* OR boy OR boys OR teen OR teens OR teenager* OR youth* OR pediatr* OR paediatr* OR puber*) NOT (adult* OR man OR men OR woman OR women))))	1,196
S4	S1 AND S2 AND S3	1,678
S3	DE "Statistical Regression" OR DE "Logistic Regression" OR DE "Severity (Disorders)" OR DE "Morbidity" OR TI ((("rate*" OR "validat*" OR "diagnos*") N3 ("tool*" OR "assess*" OR "measur*" OR "instrument*" OR "survey*" OR "questionnaire*" OR "index" OR "standard*" OR "analys*" OR "criteri*")) OR "logistic regress*" OR "regression analys*" OR "sampl*" OR "prevalen*" OR "inciden*" OR "severit*") OR AB ((("rate*" OR "validat*" OR "diagnos*") N3 ("tool*" OR "assess*" OR "measur*" OR "instrument*" OR "survey*" OR "questionnaire*" OR "index" OR "standard*" OR "analys*" OR "criteri*")) OR "logistic regress*" OR "regression analys*" OR "sampl*" OR "prevalen*" OR "inciden*" OR "severit*") OR KW ((("rate*" OR "validat*" OR "diagnos*") N3 ("tool*" OR "assess*" OR "measur*" OR "instrument*" OR "survey*" OR "questionnaire*" OR "index" OR "standard*" OR "analys*" OR "criteri*")) OR "logistic regress*" OR "regression analys*" OR "sampl*" OR "prevalen*" OR "inciden*" OR "severit*")	1,164,678
S2	DE "Mental Health" OR DE "Mental Disorders" OR DE "Affective Disorders" OR DE "Major Depression" OR DE "Anxiety Disorders" OR DE "Anxiety" OR DE "Behavior Disorders" OR DE "Bipolar Disorder" OR DE "Borderline States" OR DE "Chronic Mental Illness" OR DE "Eating Disorders" OR DE "Anorexia Nervosa" OR DE "Binge Eating Disorder" OR DE "Bulimia" OR DE "Mental Disorders due to General Medical Conditions" OR DE "Neurosis"	1,556,966

Search	Query	Results
	**OR DE "Obsessive Compulsive Disorder" OR DE "Personality Disorders" OR DE "Psychosis" OR DE "Serious Mental Illness" OR DE "Sleep Wake Disorders" OR DE "Somatoform Disorders" OR DE "Stress and Trauma Related Disorders" OR DE "Substance Related and Addictive Disorders" OR DE "Attention Deficit Disorder" OR DE "Attention Deficit Disorder with Hyperactivity" OR DE "Suicide" OR DE "Affective Psychosis" OR DE "Schizophrenia" OR DE "Schizoaffective Disorder" OR DE "Schizophrenia (Disorganized Type)" OR DE "Schizophreniform Disorder" OR DE "Undifferentiated Schizophrenia" OR DE "Substance Induced Psychotic Disorders" OR DE "Alcohol Induced Psychotic Disorders" OR DE "Disruptive Behavior Disorders" OR DE "Conduct Disorder" OR DE "Oppositional Defiant Disorder" OR DE "Autism Spectrum Disorders" OR TI ("mental" OR "psychiatric" OR "depress*" OR "anxiet*" OR "suicid*" OR "agoraphobia" OR "phobia" OR "obsessive compuls*" OR "anorexia nervos*" OR "bulimia nervos*" OR "binge-eat*" OR "eating disorder*" OR "bipolar disorder*" OR "manic disorder*" OR "personality disorder*" OR "psychotic disorder*" OR "psychos*" OR "schizo*" OR "sleep disorder*" OR "substance-related disorder*" OR "stress disorder*" OR "attention deficit" OR "disruptive behav*" OR "conduct disorder*" OR "mood disorder*" OR "autism" OR "asperger*") OR AB ("mental" OR "psychiatric" OR "depress*" OR "anxiet*" OR "suicid*" OR "agoraphobia" OR "phobia" OR "obsessive compuls*" OR "anorexia nervos*" OR "bulimia nervos*" OR "binge-eat*" OR "eating disorder*" OR "bipolar disorder*" OR "manic disorder*" OR "personality disorder*" OR "psychotic disorder*" OR "psychos*" OR "schizo*" OR "sleep disorder*" OR "substance-related disorder*" OR "stress disorder*" OR "attention deficit" OR "disruptive behav*" OR "conduct disorder*" OR "mood disorder*" OR "autism" OR "asperger*") OR KW ("mental" OR "psychiatric" OR "depress*" OR "anxiet*" OR "suicid*" OR "agoraphobia" OR "phobia" OR "obsessive compuls*" OR "anorexia nervos*" OR "bulimia nervos*" OR "binge-eat*" OR "eating disorder*" OR "bipolar disorder*" OR "manic disorder*" OR "personality disorder*" OR "psychotic disorder*" OR "psychos*" OR "schizo*" OR "sleep disorder*" OR "substance-related disorder*" OR "stress disorder*" OR "attention deficit" OR "disruptive behav*" OR "conduct disorder*" OR "mood disorder*" OR "autism" OR "asperger*")**	
S1	**DE "Hearing Disorders" OR DE "Hearing Loss" OR DE "Deafness" OR DE "Hearing Aids" OR DE "Cochlear Implants" OR TI ((("hearing") N3 ("impair*" OR "disorder*" OR "disabled" OR "loss*" OR "defect*" OR "damage" OR "handicap*" OR "function*" OR "dysfunction*")) OR "hard-of-hearing" OR "deaf*" OR "d deaf*" OR "auditory rehab*" OR "auditory disab*" OR "auditory impair*" OR "auditory handicap*" OR "hypoacus*" OR "hearing aid*" OR "hearing implant*" OR "hearing prosthes*" OR "hearing device*" OR "cochlear implant*" OR "cochlear prosthes*" OR "cochlear device*" OR (("auditory") AND ("implant*" OR "prosthes*"))) OR AB ((("hearing") N3 ("impair*" OR "disorder*"**	40,004

Search	Query	Results
	OR "disabled" OR "loss*" OR "defect*" OR "damage" OR "handicap*" OR "function*" OR "dysfunction*")) OR "hard-of-hearing" OR "deaf*" OR "d deaf*" OR "auditory rehab*" OR "auditory disab*" OR "auditory impair*" OR "auditory handicap*" OR "hypoacus*" OR "hearing aid*" OR "hearing implant*" OR "hearing prosthes*" OR "hearing device*" OR "cochlear implant*" OR "cochlear prosthes*" OR "cochlear device*" OR (("auditory") AND ("implant*" OR "prosthes*"))) OR KW ((("hearing") N3 ("impair*" OR "disorder*" OR "disabled" OR "loss*" OR "defect*" OR "damage" OR "handicap*" OR "function*" OR "dysfunction*")) OR "hard-of-hearing" OR "deaf*" OR "d deaf*" OR "auditory rehab*" OR "auditory disab*" OR "auditory impair*" OR "auditory handicap*" OR "hypoacus*" OR "hearing aid*" OR "hearing implant*" OR "hearing prosthes*" OR "hearing device*" OR "cochlear implant*" OR "cochlear prosthes*" OR "cochlear device*" OR (("auditory") AND ("implant*" OR "prosthes*")))	

Web of Science (Core Collection) Search Results – 13 December 2025

Search	Query	Results
#5	**#4 NOT TS=(("adolescen*" OR "child*" OR "schoolchild*" OR "infant*" OR "girl*" OR "boy" OR "boys" OR "teen" OR "teens" OR "teenager*" OR "youth*" OR "pediatr*" OR "paediatr*" OR "puber*") NOT ("adult*" OR "man" OR "men" OR "woman" OR "women"))**	3,297
#4	**#1 AND #2 AND #3**	4,371
#3	**TS=((("rate*" OR "validat*" OR "diagnos*") NEAR/3 ("tool*" OR "assess*" OR "measur*" OR "instrument*" OR "survey*" OR "questionnaire*" OR "index" OR "standard*" OR "analys*" OR "criteri*")) OR "logistic regress*" OR "regression analys*" OR "sampl*" OR "prevalen*" OR "inciden*" OR "severit*")**	9,882,765
#2	**TS=("mental" OR "psychiatric" OR "depress*" OR "anxiet*" OR "suicid*" OR "agoraphobia" OR "phobia" OR "obsessive compuls*" OR "anorexia nervos*" OR "bulimia nervos*" OR "binge-eat*" OR "eating disorder*" OR "bipolar disorder*" OR "manic disorder*" OR "personality disorder*" OR "psychotic disorder*" OR "psychos*" OR "schizo*" OR "sleep disorder*" OR "substance-related disorder*" OR "stress disorder*" OR "attention deficit" OR "disruptive behav*" OR "conduct disorder*" OR "mood disorder*" OR "autism" OR "asperger*")**	2,652,147
#1	**TS=((("hearing") NEAR/3 ("impair*" OR "disorder*" OR "disabled" OR "loss*" OR "defect*" OR "damage" OR "handicap*" OR "function*" OR "dysfunction*")) OR "hard-of-hearing" OR "deaf*" OR "d deaf*" OR "auditory rehab*" OR "auditory disab*" OR "auditory impair*" OR "auditory handicap*"**	149,979

Search	Query	Results
	**OR "hypoacus*" OR "hearing aid*" OR "hearing implant*" OR "hearing prosthes*" OR "hearing device*" OR "cochlear implant*" OR "cochlear prosthes*" OR "cochlear device*" OR (("auditory") AND ("implant*" OR "prosthes*")))**	

Supplement D

CLARITY tool explanation.

1. Is the source population representative of the general population?

Low – selection of target population from a representative population roster such as a national population registry

High – hospital-based patient records, undefined source population, volunteer recruitment

2. Was selection of exposed and non-exposed cohorts drawn from the same population?

Low – exposed and unexposed are drawn for same administrative database of patients presenting at same points of care over the same time frame

High – exposed and unexposed presenting to different points of care or over a different time frame

3. Can we be confident in the assessment of exposure?

Low – secure record (surgery records, pharmacy records); repeated interview or other ascertainment of current exposure

High – uncertain how exposure information obtained

4. Can we be confident in the assessment of the outcome?

Low – independent blind assessment, record linkage, validated instrument High – clinical interviews, chart diagnosis, unvalidated or ad hoc instrument

5. Did the design or analysis account for important confounding factors?

Low – comprehensive matching or adjustments for all plausible confounding factors High – matching or adjustments for few or no confounding factors

6. Can we be confident in the assessment of no confounding factors?

Low – participant interview, self-completed survey, chart review with reproducibility, database with documented accuracy

High – database with no information on accuracy of confounding factors

7. Missing data

Low – sufficient response proportion (>50%) with little missing data for variables (<15%)

High – low response proportion (<50%) and substantial missing data for variables (>15%)

Supplement E

References of the studies included in the systematic review.

Ahrenfeldt LJ, Moller S, Nielsen DL, Kjaer NK, Sondergaard J and Lykkegaard J (2024) Sensory impairments and depressive symptoms in Europe: a cross-national cohort study. *Aging & Mental Health* **28**(12)**,** 1591-1599. https://doi.org/10.1080/13607863.2024.2345790.

Akobirshoev I, McKee MM, Reif S, Adams RS, Li FS and Mitra M (2022) Opioid use disorder-related emergency department visits among deaf or hard of hearing adults in the United States. *Disability and Health Journal* **15**(2S)**,** 101291. https://doi.org/10.1016/j.dhjo.2022.101291.

Akram B and Batool M (2020) Suicidal Behavior Among the Youth With and Without Sensory Impairment: Prevalence and Comparison. *Omega* **81**(3)**,** 393-403. https://doi.org/10.1177/0030222818779711.

Aram J, Dallal CM, Cosgrove C, Arria A, Liu H and Slopen N (2024) The risk of drug overdose death among adults with select types of disabilities in the United States – A longitudinal study using nationally representative data. *Preventive Medicine* **178**. https://doi.org/10.1016/j.ypmed.2023.107799.

Awad M, Abdalla I, Jara SM, Huang TC, Adams ME and Choi JS (2024) Association of Sleep Characteristics with Tinnitus and Hearing Loss. *OTO open* **8**(1)**,** e117. https://doi.org/10.1002/oto2.117.

Bakir S, Penbegul N, Gun R, Yorgancilar E, Kinis V, Ozbay M, Atar M and Gunes M (2013) Relationship between hearing loss and sexual dysfunction. *The Journal of Laryngology and Otology* **127**(2)**,** 142-147. https://doi.org/10.1017/S0022215112002952.

Bernard A, Weiss S, Rahman M, Ulin SS, D'Souza C, Salgat A, Panzer K, Stein JD, Meade MA, McKee MM and Ehrlich JR (2022) The Impact of COVID-19 and Pandemic Mitigation Measures on Persons With Sensory Impairment. *American Journal of Ophthalmology* **234,** 49-58. https://doi.org/10.1016/j.ajo.2021.06.019.

Bigelow RT, Reed NS, Brewster KK, Huang A, Rebok G, Rutherford BR and Lin FR (2020) Association of Hearing Loss With Psychological Distress and Utilization of Mental Health Services Among Adults in the United States. *JAMA Network Open* **3**(7)**,** e2010986. https://doi.org/10.1001/jamanetworkopen.2020.10986.

Bikbov MM, Gilmanshin TR, Kazakbaeva GM, Iakupova EM, Panda-Jonas S, Zainullin RM, Fakhretdinova AA, Tuliakova AM, Gilemzianova LI, Khakimov DA, Miniazeva LA and Jonas JB (2023) Prevalence of depression, anxiety and suicidal ideas and associated factors, in particular sensory impairments, in a population of Bashkortostan in Russia. *Scientific Reports* **13**(1)**,** 17256. https://doi.org/10.1038/s41598-023-44561-1.

Bizjak MC (2009) Understanding Emotional Health and Psychological Adjustment in Students With or Without a Specific Hearing Deficiency. *Journal of Developmental and Physical Disabilities* **21**(3)**,** 213-224. https://doi.org/10.1007/s10882-009-9136-x.

Choi JW and Han E (2021) Risk of new-onset depressive disorders after hearing impairment in adults: A nationwide retrospective cohort study. *Psychiatry Research* **295,** 113351. https://doi.org/10.1016/j.psychres.2020.113351.

Ciesla K, Lewandowska M and Skarzynski H (2016) Health-related quality of life and mental distress in patients with partial deafness: preliminary findings. *European Archives of Oto-Rhino-Laryngology* **273**(3)**,** 767-776. https://doi.org/10.1007/s00405-015-3713-7.

Cormier K, Brennan C and Sharma A (2024) Hearing loss and psychosocial outcomes: Influences of social emotional aspects and personality. *PLoS One* **19**(6)**,** e0304428. https://doi.org/10.1371/journal.pone.0304428.

Cui Y, Wu T, Du H and Zhang W (2025) Exploring the association between hearing status and suicidal ideation among U.S. adults: An observational study. *General hospital psychiatry* **92,** 52-59. https://doi.org/10.1016/j.genhosppsych.2024.12.014.

Dammeyer J and Chapman M (2017) Prevalence and characteristics of self-reported physical and mental disorders among adults with hearing loss in Denmark: a national survey. *Social Psychiatry and Psychiatric Epidemiology* **52**(7)**,** 807-813. https://doi.org/10.1007/s00127-017-1397-6.

Del Vecchio V, Tricarico L, Pisani A, Serra N, D'Errico D, De Corso E, Rea T, Picciotti PM, Laria C, Manna G, Franze A, Malesci R and Fetoni AR (2023) Vascular Factors in Patients with Midlife Sensorineural Hearing Loss and the Progression to Mild Cognitive Impairment. *Medicina (Kaunas, Lithuania)* **59**(3)**,** 481. https://doi.org/10.3390/medicina59030481.

Fellinger J, Holzinger D, Dobner U, Gerich J, Lehner R, Lenz G and Goldberg D (2005) Mental distress and quality of life in a deaf population. *Social Psychiatry and Psychiatric Epidemiology* **40**(9)**,** 737-742. https://doi.org/10.1007/s00127-005-0936-8.

Fors A, Abel KM, Wicks S, Magnusson C and Dalman C (2013) Hearing and speech impairment at age 4 and risk of later non-affective psychosis. *Psychological Medicine* **43**(10)**,** 2067-2076. https://doi.org/10.1017/S0033291712002644.

Fox ML, James TG and Barnett SL (2020) Suicidal Behaviors and Help-Seeking Attitudes Among Deaf and Hard-of-Hearing College Students. *Suicide and Life-Threatening Behavior* **50**(2)**,** 387-396. https://doi.org/10.1111/sltb.12595.

Gao J, Zhu D, Deal JA, Lin FR and He P (2022) Hearing impairment, family financial support, and depressive symptoms among Chinese middle-aged and older adults. *International Journal of Geriatric Psychiatry* **37**(9)**,** 1-10. https://doi.org/10.1002/gps.5788.

Genc GA, Muluk NB and Belgin E (2013) The effects of tinnitus and/or hearing loss on the Symptom Checklist-90-Revised test. *Auris Nasus Larynx* **40**(2)**,** 154-161. https://doi.org/10.1016/j.anl.2012.06.002.

Grewal M and Golub J (2023) Association Between Hearing Loss and Multiple Negative Emotional States in the US Hispanic Population. *Otolaryngology–Head and Neck Surgery* **168**(5)**,** 1047-1053. https://doi.org/10.1002/ohn.208.

Hou X, Yang C, Wan M and Yang L (2025) When love becomes a hurt: Parental overprotection and suicide risk among deaf and hearing college students. *Current Psychology***,** 18714-18724. https://doi.org/10.1007/s12144-025-08453-z.

Hsu WT, Hsu CC, Wen MH, Lin HC, Tsai HT, Su P, Sun CT, Lin CL, Hsu CY, Chang KH and Hsu YC (2016) Increased risk of depression in patients with acquired sensory hearing loss: A 12-year follow-up study. *Medicine (Baltimore)* **95**(44)**,** e5312. https://doi.org/10.1097/MD.0000000000005312.

Idstad M, Tambs K, Aarhus L and Engdahl BL (2019) Childhood sensorineural hearing loss and adult mental health up to 43 years later: results from the HUNT study. *BMC Public Health* **19**(1), 168. https://doi.org/10.1186/s12889-019-6449-2.

Keidser G, Seeto M, Rudner M, Hygge S and Ronnberg J (2015) On the relationship between functional hearing and depression. *International Journal of Audiology* **54**(10), 653-664. https://doi.org/10.3109/14992027.2015.1046503.

Kushalnagar P, Bruce S, Sutton T and Leigh IW (2017) Retrospective Basic Parent-Child Communication Difficulties and Risk of Depression in Deaf Adults. *Journal of Developmental and Physical Disabilities* **29**(1), 25-34. https://doi.org/10.1007/s10882-016-9501-5.

Kushalnagar P, Reesman J, Holcomb T and Ryan C (2019) Prevalence of Anxiety or Depression Diagnosis in Deaf Adults. *Journal of Deaf Studies and Deaf Education* **24**(4), 378-385. https://doi.org/10.1093/deafed/enz017.

Lee DJ and Gomez-Marin O (1997) Major depressive disorder, depressive symptoms, and bilateral hearing loss in hispanic adults. *Journal of Affective Disorders* **44**(2-3), 189-195. https://doi.org/10.1016/s0165-0327(97)00044-x.

Leigh IW, Robins CJ, Welkowitz J and Bond RN (1989) Toward greater understanding of depression in deaf individuals. *American Annals of the Deaf* **134**(4), 249-254. https://doi.org/10.1353/aad.2012.0662.

Li CM, Zhang X, Hoffman HJ, Cotch MF, Themann CL and Wilson MR (2014) Hearing impairment associated with depression in US adults, National Health and Nutrition Examination Survey 2005-2010. *JAMA Otolaryngology – Head & Neck Surgery* **140**(4), 293-302. https://doi.org/10.1001/jamaoto.2014.42.

Linszen MMJ, van Zanten GA, Teunisse RJ, Brouwer RM, Scheltens P and Sommer IE (2019) Auditory hallucinations in adults with hearing impairment: a large prevalence study. *Psychological Medicine* **49**(1), 132-139. https://doi.org/10.1017/S0033291718000594.

MacGregor AJ, Joseph AR and Dougherty AL (2023) Self-Reported Hearing Aid Requirements among U.S. Military Personnel and the Association with Probable Posttraumatic Stress Disorder. *Journal of the American Academy of Audiology* **34**(7), 170-175. https://doi.org/10.1055/s-0044-1789601.

Marlow NM, Xie Z, Tanner R, Jo A and Kirby AV (2021) Association Between Disability and Suicide-Related Outcomes Among U.S. Adults. *American Journal of Preventive Medicine* **61**(6), 852-862. https://doi.org/10.1016/j.amepre.2021.05.035.

McClintock HF, Hinson-Enslin AM and Nahhas RW (2023) Depression as a mediator of the association between vision and/or hearing loss and recent substance use: Nhanes 2013–2018. *Disability and Health Journal*, 101575. https://doi.org/10.1016/j.dhjo.2023.101575.

McDermott S, Moran R, Platt T, Issac T, Wood H and Dasari S (2005) Depression in adults with disabilities, in primary care. *Disability and Rehabilitation* **27**(3), 117-123. https://doi.org/10.1080/09638280400007380.

McKee MM, Meade MA, Zazove P, Stewart HJ, Jannausch ML and Ilgen MA (2019) The Relationship Between Hearing Loss and Substance Use Disorders Among Adults in the U.S. *American Journal of Preventive Medicine* **56**(4), 586-590. https://doi.org/10.1016/j.amepre.2018.10.026.

Meher T and Gharge S (2022) Visual and hearing impairment and their association with depression among middle-aged and older individuals in India: Evidence from a cross-sectional study. *International Journal of Geriatric Psychiatry* **37**(5)**,** 1-12. https://doi.org/10.1002/gps.5716.

Merten N, Schultz AA, Walsh MC, van Landingham SW, Peppard PE, Ryff CDS and Malecki KC (2023) Psychological distress and well-being among sensory impaired individuals during COVID-19 lockdown measures. *Annals of Epidemiology* **79,** 19-23. https://doi.org/10.1016/j.annepidem.2023.01.002.

Park EY, Nelson-Bakkum ER, Schultz AA and Dillard LK (2024) Self-reported hearing loss and health during a pandemic: Findings from a cross-sectional analysis using a 2021 household survey. *Disability and Health Journal***,** 101706. https://doi.org/10.1016/j.dhjo.2024.101706.

Parker FNH, Fear NT, Stevelink SAM and Rafferty L (2022) Self-reported auditory problems are associated with adverse mental health outcomes and alcohol misuse in the UK Armed Forces. *Social Psychiatry and Psychiatric Epidemiology* **57**(3)**,** 563-573. https://doi.org/10.1007/s00127-021-02169-8.

Parker M, Duran B, Rhew I, Magarati M, Larimer M and Donovan D (2021) Risk and Protective Factors Associated with Moderate and Acute Suicidal Ideation among a National Sample of Tribal College and University Students 2015-2016. *The Journal of Rural Health* **37**(3)**,** 545-553. https://doi.org/10.1111/jrh.12510.

Peñacoba C, Garvi D, Gomez L and Alvarez A (2020) Psychological Well-Being, Emotional Intelligence, and Emotional Symptoms in Deaf Adults. *American Annals of the Deaf* **165**(4)**,** 436-452. https://doi.org/10.1353/aad.2020.0029.

Pojatić D and Degmecic D (2019) Symptoms of Anxiety, Depression and Attitudes Toward Justice in the World in a Sample of Deaf and Blind Persons in Osijek Baranja Region. *Socijalna Psihijatrija* **47**(2)**,** 168-184. https://doi.org/10.24869/spsih.2019.168.

Reavis KM, Snowden JM, Henry JA, Gallun FJ, Lewis MS and Carlson KF (2021) Blast exposure and self-reported hearing difficulty in service members and veterans who have normal pure-tone hearing sensitivity: The mediating role of posttraumatic stress disorder. *Journal of Speech, Language, and Hearing Research* **64**(11)**,** 4458-4467. https://doi.org/10.1044/2021_jslhr-20-00687.

Saperstein AM, Meyler S and Medalia A (2023) Hearing Loss Among People With Schizophrenia: Implications for Clinical Practice. *Psychiatric Services* **74**(5)**,** 543-546. https://doi.org/10.1176/appi.ps.20220226.

Schenkel LS, Rothman-Marshall G, Schlehofer DA, Towne TL, Burnash DL and Priddy BM (2014) Child maltreatment and trauma exposure among deaf and hard of hearing young adults. *Child Abuse & Neglect* **38**(10)**,** 1581-1589. https://doi.org/10.1016/j.chiabu.2014.04.010.

Scinicariello F, Przybyla J, Carroll Y, Eichwald J, Decker J and Breysse PN (2019) Age and sex differences in hearing loss association with depressive symptoms: analyses of NHANES 2011-2012. *Psychological Medicine* **49**(6)**,** 962-968. https://doi.org/10.1017/S0033291718001617.

Shin HY and Hwang HJ (2017) Mental Health of the People with Hearing Impairment in Korea: A Population-Based Cross-Sectional Study. *Korean Journal of Family Medicine* **38**(2)**,** 57-63. https://doi.org/10.4082/kjfm.2017.38.2.57.

Shiue I (2016) Chronic diseases and life events accounted for 2-18 % population attributable risks for adult hearing loss: UK Adult Psychiatric Morbidity Survey, 2007. *European Archives of Oto-Rhino-Laryngology* **273**(1)**,** 93-103. https://doi.org/10.1007/s00405-015-3504-1.

Stam M, Smit JH, Twisk JW, Lemke U, Smits C, Festen JM and Kramer SE (2016) Change in Psychosocial Health Status Over 5 Years in Relation to Adults' Hearing Ability in Noise. *Ear and Hearing* **37**(6)**,** 680-689. https://doi.org/10.1097/AUD.0000000000000332.

Stefanis N, Thewissen V, Bakoula C, van Os J and Myin-Germeys I (2006) Hearing impairment and psychosis: a replication in a cohort of young adults. *Schizophrenia Research* **85**(1-3)**,** 266-272. https://doi.org/10.1016/j.schres.2006.03.036.

Suzuki J, Kobayashi Y, Takahashi H, Tozuka H, Takai S, Ikeda R, Tabuchi T and Katori Y (2025) Relationship between Subjective Hearing Loss and Work-related and Somatic Issues in the Working-age Population: A Large-scale Internet-based Cross-sectional Study. *JMA journal* **8**(3)**,** 753-765. https://doi.org/10.31662/jmaj.2024-0430.

Test T, Canfi A, Eyal A, Shoam-Vardi I and Sheiner EK (2011) The Influence of Hearing Impairment on Sleep Quality Among Workers Exposed to Harmful Noise. *Sleep* **34**(1)**,** 25-30. https://doi.org/DOI 10.1093/sleep/34.1.25.

Thewissen V, Myin-Germeys I, Bentall R, de Graaf R, Vollebergh W and van Os J (2005) Hearing impairment and psychosis revisited. *Schizophrenia Research* **76**(1)**,** 99-103. https://doi.org/10.1016/j.schres.2004.10.013.

Tonelli M, Wiebe N, Lunney M, Donald M, Howarth T, Evans J, Klarenbach SW, Nicholas D, Boulton T, Thompson S, Schick Makaroff K, Manns B and Hemmelgarn B (2023) Associations between hearing loss and clinical outcomes: population-based cohort study. *eClinicalMedicine* **61,** 102068. https://doi.org/10.1016/j.eclinm.2023.102068.

Tremblay KL, Pinto A, Fischer ME, Klein BE, Klein R, Levy S, Tweed TS and Cruickshanks KJ (2015) Self-Reported Hearing Difficulties Among Adults With Normal Audiograms: The Beaver Dam Offspring Study. *Ear and Hearing* **36**(6)**,** e290-299. https://doi.org/10.1097/AUD.0000000000000195.

van der Werf M, Thewissen V, Dominguez MD, Lieb R, Wittchen H and van Os J (2011) Adolescent development of psychosis as an outcome of hearing impairment: a 10-year longitudinal study. *Psychological Medicine* **41**(3)**,** 477-485. https://doi.org/10.1017/S0033291710000978.

Viertio S, Perala J, Saarni S, Koskinen S and Suvisaari J (2014) Hearing loss in persons with psychotic disorder--findings from a population-based survey. *Schizophrenia Research* **159**(2-3)**,** 309-311. https://doi.org/10.1016/j.schres.2014.08.016.

Wallace S, Mactaggart I, Banks LM, Polack S and Kuper H (2020) Association of anxiety and depression with physical and sensory functional difficulties in adults in five population-based surveys in low and middle-income countries. *PLoS One* **15**(6)**,** e0231563. https://doi.org/10.1371/journal.pone.0231563.

Supplement F

All characteristics

Study, first author, year	Disorder(s)	Type of outcome	Method	Recruitment	Country	DHH group (N)	Type of audiometric test	Hearing group (N)	Age range (if reported M (SD))	Gender/sex distribution of the sample	Language used by DHH	hearing device	DHH before or after Language acquisition	Tinnitus	Education DHH	Outcome Measure	Analysis	covariates	Effect direction - Summary	Effect direction - Majority	Result / effect size / Statistic	Risk of Bias (total)
Ahrenfeldt, 2024	Depression	Prevalence	Prospective cohort	Probability-based, population-representative sampling	European countries: Denmark, Sweden, Austria,<br>Germany, the Netherlands, France, Switzerland, Belgium,<br>Luxembourg, Spain, Italy, Greece, Portugal, Czech Republic, Poland, Hungary,<br>Slovenia, Estonia, and Croatia.	People who reported fair or poor hearing (using a hearing aid as usual) (whole sample: N = 11971)	Self-report	People who reported excellent, very good, or good hearing (using a hearing aid as usual) (whole sample: N = 86051)	50 to 64 years (NR)	Male: 48,74%, women: 51,26%, %(whole sample)	NR	Hearing ratings using hearing aid as usual	NR	NR	NR	EURO-D	Mixed effects logistic regression	Sex, age, partner in household, education, household income, and history of diseases including heart attack, hypertension, stroke, diabetes, Parkinson's disease, cancer, arthritis, and lung disease.	Higher in DHH	Higher in DHH	AOR: 1.28 (1.13-1.45)	Lower
Akobirshoev, 2022	Psychotic disorders<br>Depression and Anxiety pooled<br>Substance use disorders (opioid, alcohol)	Prevalence	Cross-sectional	Hospital	USA	Deaf and hard of hearing (N = 63865)	ICD-10-CM codes H90xx and or H91xx in register/archival data	No record of DHH, age-matched, sex-matched, and year-matched (191595)	18-64 (46.42 (0.14))	Men: 50.6%, women: 49.4%	NR	NR	NR	NR	NR	ICD-10-CM codes, Opioid = emergency department visits	Odds ratio, Opioid = Multiple logistic regression	None, opioid = sociodemographic and hospital characteristics; clinical characteristics; chronic pain.	Higher in DHH	Higher in DHH	Psychotic disorders = OR: 2.3483 (2.2607 to 2.4394)<br>Depression and Anxiety pooled = OR: 2.6039 (2.5445 to 2.6647)<br>Substance use disorders (opioid = AOR 1.22 (1.14-1.30), alcohol = OR: 1.2514 (1.2033 to 1.3014)	Lower
Akram, 2020	Suicidal outcomes	Prevalence	Cross-sectional	Education institutions	Pakistan	Hearing impairment (N = 535)	NR	Without sensory impairment (N = 595)	DHH: 18.4 (2.01)	Men: 54,16%, women: 45,84%	NR	NR	NR	NR	NR	Suicidal Behavior Questionnaire Revised (SBQ-R)	Binary logistic regression	None	Higher in DHH	Higher in DHH	OR: 2.01 (1.91 to 3.16)	Higher
Aram, 2024	Substance use disorders	Prevalence	Retrospective cohort	Nationally-representative survey linked to National Death index	USA	Hearing limitation (deaf or serious difficulty hearing) (N = 82500)	Self-report	Adults without a disability (N = 2802000)	> 18	Male: 48.3%, female: 51,7%	NR	NR	NR	NR	NR	Drug overdose deaths are identified using ICD-10 underlying cause of death<br>codes X40–44, X60–64, X85, and Y10-Y14	Cox proportional hazards regression models	Age, sex, race/ethnicity, marital status, education, employment, poverty	Higher in DHH	Higher in DHH	AHR = 1.6 (1.3 to 1.9)	Lower
Awad, 2024	Sleep-wake disorders	Prevalence	Cross-sectional	National survey	USA	Hearing loss >25 dB (Ntotal = 9693)	PTA	Hearing loss < 25 dB (Ntotal = 9693)	> 20 (51,7 (18,7))	Male: 50,7%, female: 49.3%	NR	NR	NR	NR	NR	Self-report: had ever been told by a doctor that they had a sleep disorder	Multivariable logistic regression	Age , sex, race, education, income, health insurance, medical history, and noise exposure.	No difference	No difference	AOR: 1.08 (0.67-1.74)	Lower
Bakir, 2013	Sexual Dysfunctions	Symptom severity	Prospective case–control	ENT	Turkey	Hearing loss was mild in 13 (36.1 per cent) patients, moderate in 15 (41.7 percent) and moderately severe in eight (22.2 per cent), according to the American Speech-Language-Hearing Association guidelines<br>(N total = 36)	PTA	Healthy, adult, married men who were demonstrated to have normal hearing levels. (N = 40)	24 to 57 (DHH: 37.8 (8.7))	100% men	NR	NR	after	NR	NR	International Index of Erectile Function questionnaire.	T-test	None	Higher in DHH	Higher in DHH	Total score, HL: M (SD) = 49.8 ± 6.7, Control: M (SD) = 59.7 ± 10.5, p < .001	Higher
Bernard, 2022	Depression	Symptom severity	Cross-sectional	Clinical setting	USA	People with hearing impairment<br>(N = 108)	ICD-10 codes corresponding to a diagnosis of hearing loss or deafness	Not hearing/visually impaired<br>(N = 155)	60% <50 - 65 years old	Male: 51,33%, female: 48,67%	Speaking and understanding English	NR	NR	NR	NR	Patient Health Questionnaire (PHQ-2)	1-way analysis of variance (ANOVA)	Age, sex, marital status, comorbidity index (CCI), area deprivation index	No difference	No difference	DHH M = 1.4 (SD = 1.66), Control: M = 1.63 (SD = 1.79), p = 0.23	Lower
Bigelow, 2020	Depression<br>Anxiety<br>General mental disorders	Prevalence	Cross-sectional	Nationally representative survey	USA	Moderate or worse hearing loss (N = 1526, general mental disorders N = 2247)	Self-report	No hearing loss (N = 6743, general mental disorders N = 9806)	18 to 65	Male: 48.3%	NR	No HL: 0.09% used hearing aid, moderate or worse HL: 20.6% used hearing aid no significant difference in depression.	NR	NR	NR	Antidepressant medication use<br>Antianxiety medication use<br>Kessler 6	Multiple logistic regression	Age, sex, race, family income, education, marital status, self-reported health, heart disease, hypertension, diabetes, stroke, and smoking history.	Higher in DHH	Higher in DHH	Depression = AOR: 2.18 (1.69-2.81)<br>Anxiety = AOR: 1.91 (1.49-2.44)<br>General mental disorders = Moderate General severity = AOR: 2.12 (1.83-2.47), Serious general severity = AOR: 2.25 (1.73-2.93)	Lower
Bikbov, 2023	Depression<br>Anxiety<br>Suicidal outcomes	Symptom severity<br>Prevalence	Cross-sectional	Community	Russia	Hearing loss score range (N = 1538)	Self-report questionnaire	Hearing loss score range<br>(N = 4355)	40 to 94 (59 (NR))	Women: 56,3%	NR	NR	Nr	NR	NR	Center for Epidemiologic Studies Depression Scale (CES-D)<br>State Trait Inventory Anxiety Test<br>Self-reported suicidal ideas	Multiple linear regression<br>Multiple logistic regression	20 variables (e.g. sex, age, level of education, number of days with fruit intake, prevalence of reported thoracic spine pain, unconsciousness, vigorous activity in leisure time, hand grip force, prevalence of dry eye disease)	Higher in DHH	Higher in DHH	Depression = B = 0.02 (0.01 to 0.03)<br>Anxiety = B = 0.02 (0.01 to 0.03)<br>Suicidal outcomes = AOR: 1.02 (1.00 - 1.04)	Lower
Bizjak, 2009	Sexual Dysfunctions	Symptom severity	Cross-sectional	Deaf schools / local schools	Slovenia	prelingual hearing loss, severe (>65dB) or profound (>90dB) (N = 36)	NR	Hearing sample, without hearing difficulties (N = 36)	16 to 21	Boys: 38,89%, girls: 61,11%	Primarily sign	NR	Prelingual HL, (all of them were born deaf or lost their hearing before the age of 4 years)	NR	Special	The Trauma Symptom Checklist for Children (TSCC)	Mann-Whitney U test	None	No difference	No difference	Depression = DHH mean (SD) 5.75 (3.36) control 6.53 (4.5), p = .671<br>Anxiety = DHH mean (SD) 6.25 (3.23) control 6.58 (3.88), p = .946<br>Sexual health = Sexual concerns = DHH M (SD) 7.19 (4.38), TH = M*SD) 7.83 (5.09), p = .680.<br>Sexual distress = DHH M (SD) 2.64 (1.98), TH M (SD) = 2.31 (2.21), p = 0.350	Higher
Choi, 2021	Depression	Incidence	Retrospective cohort	Register	South-Korea	Hearing impairment was defined as having a hearing threshold ≥60 dB in both ears or ≥80 dB in one ear and ≥40 dB in the other.<br>(N = 832)	PTA	Propensity score matched sample of no hearing impairment (N = 2494)	20 to 59	Male: 52,5%, female: 47,5%	NR	NR	NR	NR	NR	Insurance depressive disorder (ICD-10 code: F32–F33)	Cox proportional hazard models	Propensity score matched on sex, age, index year, comorbidities, history of depression, household income, insurance type, and residential area.	No difference	No difference	Middle-aged adults, new onset = HR: 1.15 (0.91 - 1.42); Middle-aged adults, recurrent = HR: 0.86 (0.54 - 1.32); Young adults, new onset = HR: 0.62 (0.31 - 1.27); Young adults, new onset = HR: 0.93 (0.27 - 3.01)	Lower
Ciesla, 2016	Depression<br>Anxiety	Symptom severity	Cross-sectional	ENT	Poland	Bilateral symmetrical sensorineural hearing loss, mean PTA (dB) prelingual: 71.2 (SD = 19.87, range: 23 -97)), mean PTA (dB) post lingual: 57.3 (SD = 21.11, range 20 - 97). (N = 31)	PTA	Typical hearing (N = 31)	18,5 to 53,8 (DHH: 37.6 (7.9))	Men: 48,39%, women: 51,61%	All patients had well developed verbal skills and used auditory verbal communication.	Nineteen patients were regular users of either one (10 patients) or two optimally fitted hearing devices (9 patients) and 12 patients were non-users (as they had no significant gain)	There were 14 patients with a prelingual hearing loss (developed and diagnosed before the age of 3 years) and 17 patients with a post lingual hearing loss (developed and diagnosed after the age of 12 years)	16 yes, 15 no	NR	Beck Depression Inventory (BDI)<br>State-Trait-Anxiety-Inventory (STAI)	Two sample t-test	None	Higher in DHH	Higher in DHH	Depression = t(60) = 2.77; p = 0.007<br>Anxiety = STAI-State = t (60) = 2.49; p = 0.016; STAI-Trait = t (60) = 2.50; p=0.015.	Higher
Cormier, 2024	Depression<br>Anxiety	Symptom severity	Cross-sectional	Community	USA	Hearing loss (N = 267) (moderate hearing loss (41%), severe hearing loss (34%), mild hearing loss (23%), and 2% unknown severity).	Self-report	No hearing loss (N = 501)	> 18 (≈ 50 (18))	Male: 24%, female: 76%, other: 0.78%	NR	22 CI users and 139 hearing aid users completed this survey. One hundred and fourteen individuals in this survey with self-reported hearing loss did not utilize a hearing device.	NR	NR	NR	The Patient Health Questionnaire-4 (PHQ-4)	Serial ordinary least squares regression models	Demographic, psychosocial, social emotional, and personality trait variables were added to the controlled regression. Interaction terms were included between hearing loss variables and age, psychosocial, social emotional, personality traits, and tinnitus.	No difference	No difference	Depression = b = - 0.04 (-0.14 to 0.05)<br>Anxiety = b = 0.02 (-0.08 to 0.11)	Higher
Cui, 2025	Suicidal outcomes	Prevalence	Cross-sectional	National survey	USA	A little trouble hearing (N = 1197), Moderate trouble hearing (N = 511), Deaf or Hard of hearing (N = 229) (NDHH = 1937)	Self-report	Excellent (N = 2923) or good hearing (N = 3235) (Nhearing = 6158)	> 20 (48,03 (17,02))	Male: 51,66%, female: 48,34%	NR	NR	NR	NR	NR	Asked participants how often, over the past two weeks, they had experienced thoughts that they would be better off dead or had thoughts of hurting themselves.	Multiple logistic regression	Age, genders, race, education levels, marital status, family monthly poverty level, BMI, NHHR, vision trouble, mobility trouble, smoke, hypertension, diabetes, coronary heart disease, stroke, cancer.	Higher in DHH	Higher in DHH	A little trouble AOR: 2.40(1.12,5.16), Moderate hearing trouble AOR: 2.95(1.20,7.22), Deaf or Hard of hearing AOR: 5.72 (1.73,10.24)	Lower
Dammeyer, 2017	General mental disorders<br>Intellectual developmental disorder	prevalence	Cross-sectional	Community	Denmark	Estimated 25% of the Danish population with profound hearing loss; range from Moderate (40 - 54dB) to profound (>90dB) (N = 774)	Self-report	General population (N = 18017)	16 to 65 (DHH: 43.1 (14.3))	Men: 46,84%	Speech: 62,10%, sign: 26,26%, other: 11,64%	NR	51,10% diagnosed before the age of 4	NR	NR	Self-reported diagnosis of a mental disorder/disability	Fisher's exact test or Chi square statistics for categorical variables.	None	Higher in DHH	Higher in DHH	One mental disorder = OR: 1.6778 (1.3139 to 2.1425), more than one mental disorder = OR: 3.5596 (2.5720 to 4.9263).<br>intellectual developmental disorder = OR: 70.37 (14.18 to 349.23)	Higher
Del Vecchio, 2023	Depression<br>Anxiety	Symptom severity	Retrospective case-control	Audiology clinics	Italy	Bilateral SNHL, mild HL (58; 71.5%) as 26 to 40 dB HL, moderate HL 9 (11.9%) as 41 to 55 dB HL, moderately severe HL (6; 7.4%) as 56 to 70 dB HL, severe HL (6; 7.4%) as 71 to 90 dB HL, and profound HL (2; 2.5%) as 91 dB HL or worse.<br>(N = 81)	Otoscopy, tympanometry and acoustic reflex measurement, standard PTA, bone conduction hearing	Normal hearing in the absence of subjective HL, tinnitus, and fullness. (N = 31)	50 to 65 (DHH: 58 (5.2))	Men: 72.3%, women: 27.7%	NR	NR	NR	48/81 (59.3%) of patients in the SG experienced a continuous tinnitus	NR	Hospital Anxiety and Depression Scale - Depression/Anxiety (HADS-D/A)	Mann-Whitney U test	None	No difference	No difference	Depression = DHH median: 4 (1.25, 8); TH: median: 2 (0.75, 5.25) p = 0.26.<br>Anxiety = median: 6 (4, 9.75) in DHH, 5 (1.75, 7,25) in TH, p = 0.64	Higher
Fellinger, 2005	Depression<br>Anxiety<br>Psychotic disorders<br>General mental disorders<br>Somatic Symptom disorder	Symptom severity	Cross-sectional	The association of the deaf	Austria	Members from the deaf community<br>(N = 233)	NR	German general population<br>(N = 600)	NR (45.3 (14.4))	Male: 51,74%, Female: 48,26%	NR	NR	NR	NR	95.3% had attended schools for the deaf	Brief Symptom Inventory (BSI)	Two-sample t-tests	None	Higher in DHH	Higher in DHH	Depression = DHH (M = 0.87 SD = 0.69); TH (M = 0.28, SD = 0.37) P < 0.01<br>Anxiety = DHH mean (SD) 0.98 (0.70); TH (SD) 0.34 (0.34) p < 0.01<br>Psychotic disorders = DHH mean (SD) 1.10 (0.77); TH mean (SD) 0.34 (0.39), p < 0.01<br>General mental disorders = DHH mean (SD) 4.38 (2.53); TH = 1.16 (2.10), p < 0.01<br>Somatic Symptom disorder = DHH mean (SD) 0.81 (0.67); TH mean (SD) 0.2 (0.32) P < 0.01	Higher
Fors, 2013	Psychotic disorders	Prevalence	Retrospective case-control	Register	Sweden	Hearing impairment at age 4, >25dB (N = 10)	PTA	Normal hearing<br>(N = 308)	18 to 28 (23.7 (NR))	Male: 55.9%, female: 44.1%	NR	NR	Hearing impairment before the age of 4	NR	NR	Non-affective psychosis (ICD-10 F20–29 or ICD-9 295, 297 and 298, excluding 298A).	Conditional logistic regression.	Parental psychiatric history, occupational class and obstetric complications, and deleted 3 individuals with both speech and hearing impairment	Higher in DHH	Higher in DHH	AOR: 6,7 (1,3 to 33,8)	Lower
Fox, 2020	Suicidal outcomes	Prevalence	Cross-sectional	Universities	USA	Deaf/hard of hearing University students (N = 5955)	Self-report	Hearing (N = 6018)	18 to 24 (DHH: 20.4 (1.7))	Male: 34.2%, female: 65.2%	NR	NR	NR	NR	NR	Self-reported "seriously considered suicide" and "attempted suicide"	Multiple logistic regression	Gender, age, race, mental health treatment/diagnosis,	Higher in DHH	Higher in DHH	Ideation = past year (AOR = 1.91; 95% CI: 1.69 to 2.16), attempt past year = (AOR = 2.42; 95% CI: 1.85 to 3.17), more than 12 months ago (AOR = 1.49; 95% CI: 1.34,1.65), Attempt = more than 12 months ago (AOR = 1.74; 95% CI: 1.53 to 1.98).	Higher
Gao, 2022	Depression	Prevalence	Cross-sectional	Nationally representative longitudinal survey	China	Hearing impairment (N = 2958)	Self-report	No hearing impairment (N = 17326)	>45 (Total sample wave 1: 59.12 (9.7), wave 2: 59.62 (9.53), wave 3: 59.87 (9.74))	Female: (wave 1) 52,42%; (wave 2) 52,17%; (wave 3) 51,37%	NR	NR	NR	NR	NR	Center for Epidemiologic Studies Depression Scale (CES-D)	Fixed effect logit models	Age, marital status, care support, emotional support, smoking, drinking, disability status, visual impairment, cognitive functioning, hypertension, diabetes, financial support, interactions	Higher in DHH	Higher in DHH	AOR: 1.41 (1.09-1.84)	Lower
Genç, 2013	Depression<br>Anxiety<br>Psychotic disorders<br>General mental disorders<br>Somatic Symptom disorder<br>Obsessive compulsive disorder	Symptom severity	Cross-sectional	Unclear	Turkey	Patients with hearing loss with and without tinnitus<br>(N = 68)	PTA and bone conduction hearing thresholds.	No hearing loss, with and without tinnitus<br>(N = 74)	18 to 73 (DHH: 44.78 (11.81))	Male: 48,59%, female: 51,31%,	NR	NR	Nr	70 (49,29%) had tinnitus, they controlled for tinnitus status in the analysis.	NR	SCL-90-R	Multiple linear regression	Age, gender, tinnitus presence, tinnitus duration	No difference	No difference	Depression = β = -0.127 p = 0.268<br>Anxiety = β = -0.031 p = 0.801, phobic anxiety = β = 0.168 p = 0.169<br>Psychotic disorders = paranoid ideation = β = -0.019 p = 0.870, psychoticism = β = -0.199 p = 0.105<br>General mental disorders = general strain = β = 0.059 p = 0.576, global severity = β = 0.023 p = 0.842<br>Somatic Symptom disorder = β = 0.72 p = 0.519<br>Obsessive compulsive disorder = β = -0.041 p = 0.697	Lower
Grewal, 2023	Anxiety	Symptom severity	Cross-sectional	Community	USA	Range of hearing loss (N total = 4404)	PTA	Range of hearing loss (N total = 4404)	18 to 75 (46.7 (13.7))	Male: 39.4%, female: 60.6%	NR	NR	NR	NR	NR	The Spielberger Trait Anxiety Scale	Multiple linear regression	Age, gender, and education	Higher in DHH	Higher in DHH	b = 0.41 (0.23 to 0.60) p <.01	Lower
Hou, 2025	Depression<br>Suicidal outcomes	Symptom severity	Cross-sectional	Colleges / universities	China	Deaf college students (n = 217) . 90dB (N = 31),70 - 90dB (N = 48) 60 - 70 dB (N = 52) 50 - 60 dB (N = 86).	Hospital with the national disability determination<br>Qualification	Hearing college students (N = 232)	NR (20,85 (1,7))	Male: 46,1%, female: 53,9%	NR	NR	NR	NR	NR	Depression, Anxiety, and Stress Scales (DASS-21)<br>Suicidal Behaviors Questionnaire (SBQ-R)	Multiple analysis of variance (MANOVA)	none	Higher in DHH	Higher in DHH	Depression = DHH: M = 4.63, SD = 3.59, hearing: M = 2.57, SD = 3.03, F = 42,72, p < 0.001<br>Suicidal outcomes = DHH: M = 5.44, SD = 2.41, hearing: M = 4.87, SD = 2.43, F = 6,08 , p < 0.05	Higher
Hsu, 2016	Depression	Incidence	Retrospective cohort	National register (health insurance data)	Taiwan	Patients with acquired sensory hearing loss (SHL) (N = 2524)	Health insurance database ICD-9 codes 388.01 and 389.10-389.12	No sensory hearing loss (N = 10096)	20 to 64	Men: 61,9%, women: 38,1%	NR	NR	After	NR	NR	Health insurance database ICD-9-CM codes 296.2, 296.3, 300.4, and 311	Multivariable Cox proportional hazard regression analyses	Age, sex, and comorbidities of cirrhosis, hypertension, hyperlipidemia, diabetes mellitus, asthma, chronic kidney disease, coronary artery disease, alcohol-related illness, anxiety, COPD and stroke, and medication of steroid	Mixed	No difference	20 - 49 = HR: 2.22 (1.62, 3.06), 50 - 64 = HR: 1.21 (0.88, 1.67)	Lower
Idstad, 2019	Depression and anxiety pooled	Symptom severity	Prospective cohort	Schools	Norway	66 individuals with moderate-severe hearing loss, 66 with mild hearing loss, 220 with slight hearing loss (Total N = 352)	PTA	Normal hearing (N = 32104)	20 to 56 (36.78 (8.91)	Male: 47,08%, female: 52,92%	NR	NR	NR	NR	NR	Symptom Checklist-10 (SCL-10)	ANOVA	Sex, age, education, father's education, and mother's education	No difference	No difference	slight: b = 0.11 (-0.03 to 0.24), p = 0.128, Mild: b = 0.11 (-0.14 to 0.36) p = 0.391, Moderate-Severe: b = 0.10 (-0.16 to 0.35) p = 0.456.	Lower
Keidser, 2015	Depression	Prevalence<br>Symptom severity	Cross-sectional	National register	UK	No groups, continuous hearing loss (total N = 101099)	PTA and self-report	No groups, continuous hearing loss (total N = 101099)	39 to 70 (56.8 (NR))	Male: 49%, female: 51%	NR	Only 2.7% of the study sample reported using a HA, no effect on FH general, and FH noise, but significant predictor BERSTn on average, reported higher levels of depressive symptoms than non-users.	NR	NR	NR	Constructed self-report questionnaire	Multiple regression	Age and gender.	Higher in DHH	Higher in DHH	Prevalence = The estimated effect sizes in factor scores (factor scores per 10 dB SNR for BESRTn) are also shown for the different hearing measures. FH General (Reported functional hearing in general) effect size: 0.14 (SE = 0.007) FH Noise (Reported functional hearing in noise) effect size: 0.16 (SE = 0.006); BESRTn: (Better ear speech reception threshold in noise) effect size: 0.08 (SE = 0.02).<br>Symptom severity = The estimated effect sizes in factor scores (factor scores per 10 dB SNR for BESRTn) are also shown for the different hearing measures. FH General (Reported functional hearing in general) effect size: 0.22 (SE = 0.006) FH Noise (Reported functional hearing in noise) effect size: 0.3 (SE = 0.005); BESRTn: (Better ear speech reception threshold in noise) effect size: 0.27 (SE = 0.017).	Lower
Kushalnagar, 2017	Depression	Prevalence	Cross-sectional	Colleges	USA	Deaf/hard of hearing, mild to moderate hearing severity 21%, severe to profound 79% (N = 143)	Self-report	Hearing (N = 90)	DHH: 18 to 50 (DHH: 22 (3)).	Deaf group: male: 45,45%, female: 54,55% , control: NR	57,7% sign language, 42,3% spoken	NR	NR	NR	NR	Beck Depression Inventory-2nd revision (BDI-II)	Logistic regression	Communication difficulties, language preference,	No difference	No difference	Females AOR: 1.6 (p>0.05), Males: AOR: 0.53 (P>0.05)	Higher
Kushalnagar, 2019	Depression and anxiety pooled	Prevalence	Cross-sectional	National survey	USA	Deaf adults who use American Sign Language (ASL) (N = 1704)	NR	Hearing group (N = 3287)	NR (DHH: 48 (17))	Male: 40,57%, female: 59,43%	Language preference: 48% ASL; 52% english (and asl)	NR	NR	NR	NR	Self-reported: "Has a doctor or other health professional ever told you that you had any of the following medical conditions: depression or anxiety disorder?"	Odds ratio	Age-weighted	Higher in DHH	Higher in DHH	OR: 1.1958 (1.0421 to 1.3723)	Higher
Lee, 1997	Depression	Prevalence	Cross-sectional	Representative Hispanic survey	USA	Bilateral hearing impairment average threshold levels greater than 25 dB (N = 161)	PTA	No hearing impairment (less than 25 dB Bilateral HL) (N = 2485)	20 to 74	NR	NR	NR	NR	NR	NR	Diagnostic Interview schedule<br>Center for Epidemiologic Studies Depression Scale (CES-D)	Multivariable Logistic Regression	age, gender, marital status, employment status, and insurance status.	Mixed	No difference	Diagnostic interview schedule = Cuban Americans AOR: 0.83 (0.06 - 11.88), Mexican Americans: AOR: 0.01 (0.00 - 0.03), Puerto Rican Americans: AOR: 0.35 (0.03 - 4.47).<br>CES-D = Middle tertile = Cuban Americans AOR: 0.70 (0.16 - 3.03), Mexican Americans: AOR: 1.11 (0.51 - 2.41), Puerto Rican Americans: AOR: 2.59 (1.15 - 5.82) Upper tertile = Cuban Americans AOR: 1.44 (0.60 - 3.47), Mexican Americans: AOR: 1.28 (0.40 - 4.10), Puerto Rican Americans: AOR: 4.58 (1.95 - 10.75)	Lower
Leigh, 1989	Depression	Prevalence<br>Symptom severity	Cross-sectional	Universities / colleges	USA	Deaf, hearing loss greater than 80dB, age of onset 2 or earlier (90/102 deaf at birth) parents with normal hearing and no additional handicaps (N = 102)	NR	Hearing (N = 112)	Maximum 25	Male: 47,2%, female: 52,8%	NR	NR	Onset of deafness at age 2 or orlier (in actuality 90 out of 102 subjects reported age of onset at birth, while the remainder reported becoming deaf anywhere from 2 to 24 months of age).	NR	Deaf university	Beck Depression Inventory (BDI)	Odds ratio<br>ANOVA	Gender	Mixed	No difference	Mild depression = OR: 0.4823 (0.2719 to 0.8554), moderate depression = OR: 0.4352 (0.1270 to 1.4914), severe depression = OR: 6.5525 (0.3343 to 128.4203)<br>Symptom severity = DHH M (SD) = 10.03 (5.44), TH: M (SD) 7.71 (7.28), p <0.05	Higher
Li, 2014	Depression<br>Sleep-wake disorders	Prevalence	Cross-sectional	National survey	USA	Depression = participants who self-reported little trouble hearing 62,6% (2284), moderate trouble hearing 24,61% (898), a lot of trouble hearing 12% (438), to be Deaf 0,79% (29) (N = 3649)<br>Sleep-wake disorders = Participants who self-reported little trouble hearing N = 4162	Self-report	Depression = excellent hearing (N = 6174)<br>Sleep-wake disorders = Excellent or good hearing N = 14152	18 to >85 (49.6 (NR))	Male: 49,25%, female: 50,75%	NR	Subsample: 10.3% of the 18 to 19 year old HI people, and 36.5% of the people over 70 had ever worn a hearing aid	NR	Subsample: 10,3% of all 18 to 19 (1186) year old participants had tinnitus, of which 8.3% of the hearing group, and 37,9% of the HI group. Of people over 70, (3149) 15,5% had tinnitus, of which 11.3% of the hearing group, and 19,8% of the HI group		PHQ-9 (moderate to severe)<br>Sleep disorder if diagnosed by a physician or other health professional	Multivariate logistic regression analysis<br>Odds ratio	Model 1 was controlled for age, sex, and race/ethnicity; model 3 was controlled for age, sex, race/ethnicity, marriage, living alone, educational level, income, health insurance, body mass index, smoking, binge drinking, self-reported health status, cardiovascular disease, cancer, diabetes mellitus, hypertension, sleep disorder, and trouble seeing.	Mixed	Higher in DHH	Depression = Little trouble hearing: AOR: 1.7 (1.3 to 2.2), Moderate trouble hearing - AOR: 2.4 (1.7 to 3.2), A lot of trouble hearing - AOR: 1.5 (0.9 to 2.6), Deaf - AOR: 0.6 (0.1 to 2.6).<br>Sleep-wake disorders = OR 1.43: (1.27 to 1.61)	Higher
Linszen, 2019	Psychotic disorders	Prevalence	Cross-sectional	ENT	the Netherlands	Mild hearing impairment (N = 214; 25,81%); Moderate (N = 190; 22,92%), Severe (N = 95; 11,46%), Profound (N = 70; 8,44%), Unilateral: N = 260; 31,36%) Total N = 829	PTA loss of more than 25 dB, in at least one ear	No hearing impairment (N = 173)	18 to 92 (median hallucinations: 59,5 (NR); no hallucination: 56 (NR))	Female 47.2%	Excluded participants with potential language barrier	NR	NR	NR	NR	14-item semi-structured interview	Multivariate logistic regression analysis	Sex, tinnitus, hearing impairment localization, age	Higher in DHH	Higher in DHH	for every dB OR: 1.02 (1.01 to 1.03)	Lower
MacGregor, 2023	Post-traumatic stress disorder	Prevalence	Cross-sectional	Electronic repository of Navy and Marine Corps Periodic Health Assessments (PHAs)	USA	Military personnel self-reporting requiring hearing aids (N = 1088)	Self-report	Military personnel self-reporting not requiring hearing aids (N = 103640)	> 18 (NR)	Men: 83.17%, women: 16,93%	NR	Entire DHH group	NR	NR	NR	Primary Care PTSD Screen, which is present in the Behavioral Health Section of the PHA	Multivariable logistic regression	Age, gender, service branch, military rank, and service component.	Higher in DHH	Higher in DHH	AOR: 3.45 (3.00–3.96)	Lower
Marlow, 2021	Suicidal outcomes	Prevalence	Cross-sectional	National representative survey (NSDUH)	USA	Deaf or do you have serious difficulty hearing, no other disabilities (N = 4891)	Self-report	People without disabilities<br>(N = 163959)	80.16% was between 18 and 64	Male: 48,7% , female: 51,3%	NR	NR	NR	NR	NR	Self-report "thinking/planning/attempting to kill yourself"	Multiple logistic regression	Age, sex, race/ethnicity, marital status, education, employment status, household income, insurance status, urbanization, smoking status, drinking status, obesity, health status, number of emergency department visits, number of comorbidities, and depressive episode.	Mixed	No difference	ideation = AOR: 1.41 (1.04, 1.91), planning without attempt = AOR: 0.45 (0.17, 1.20) attempt = AOR: 0.84 (0.43, 1.66)	Lower
McClintock, 2023	Depression	Prevalence	Cross-sectional	Nationally-representative survey	USA	People with serious trouble hearing (N = 271)	Self-report	Neither serious trouble hearing, vision loss or both (N = 8723)	18 - 59 (38.6, SE = 0.2))	Male: 49,7%, female: 50,3%	NR	NR	NR	NR	NR	Patient Health Questionnaire (PHQ-9)	Weighted binary logistic regression	Age, gender, race/ethnicity, military status, education, and income	Higher in DHH	Higher in DHH	AOR: 3.090 (2.184, 4.371)	Lower
McDermott, 2005	Depression	Prevalence	Retrospective cohort	Database from family medicine physicians	USA	Deaf; regardless of use of lip reading, interpreter, or hearing aide, no partial hearing loss. (N = 36)	Medical records: ICD9: 389	Age-group stratified Patients without a disability (N = 2084)	NR (DHH: 37.6 (NR))	Female: 56,83%	NR	Deaf regardless of hearing aid	NR	NR	NR	ICD-9 codes: 300.4, 311, 296.2, 296.3 or 309.1	Odds ratio	None	No difference	No difference	OR: 0.55 (0.21 to 1.41)	Lower
McKee, 2019	Substance use disorders	Prevalence	Cross-sectional	National representative survey (NSDUH)	USA	Deaf or serious difficulty hearing, 18-34 years old = 722, 35-49 years old = 556, N total = 1278	Self-report	People who are not deaf, or have serious difficulty hearing, 18-34 years old = 45326, 35-49 years old = 21974, N total = 112626	18 to 49	18 - 34 years old = Male: 49,80%, female: 50,2%; 35 - 49 years old = male: 48,84% , female: 51,16%	NR	NR	NR	NR	NR	Self-report, meeting criteria for an alcohol use disorder, cannabis use disorder, or opioid use disorder; as well as for any SUD (non-nicotine), based on DSM-IV criteria	Multiple logistic regression	Sex, race (white/black/all other), income (<$20,000, $20,000 –$49,999, ≥$50,000), health insurance, employment (full/part time, unemployed, all others), and prescription medication use for mental health in the past year	Mixed	Higher in DHH	Any substance 18-34 years old = AOR: 1.26 (1.03, 1.54), alcohol 18-34 years old = AOR: 1.12 (0.84, 1.49)<br>marijuana 18-34 years old =<br>AOR: 1.34 (0.91, 1.97)<br>prescription opioid use 18-34 years old = AOR: 2.57 (1.61, 4.11)<br>any substance 35-49 years old = AOR: 1.83 (1.36, 2.45)<br>alcohol 35-49 years old = AOR: 1.89 (1.43, 2.50)<br>marijuana 35-49 years old = AOR: 1.04 (0.57, 1.88)<br>prescription opioid use 35-49 years old = AOR: 1.98 (1.02, 3.86)	Lower
Meher, 2022	Depression	Prevalence	Cross-sectional	National representative survey	India	Hearing impairment<br>(N = 4524)	Self-report	No hearing impairment<br>(N = 61038)	> 45	Male: 45,9%, female: 54,1%	NR	NR	NR	NR	NR	Composite International Diagnostic Interview Short Form (CIDI-SF)	Multivariable Logistic regression	Age group, gender, marital status, education, working status, caste, religion, MPCE quintile, place of residence, region, smoking status, alcohol consumption, BMI and multimorbidity.	Higher in DHH	Higher in DHH	AOR: 1.25 (1.11-1.41)	Lower
Merten, 2023	Depression<br>Anxiety	Prevalence	Cross-sectional	Representative state cohort from probability-based cluster sampling	USA	Hearing impairment (fair and poor) (N = 144)	Self-report: hearing (with hearing aid, if used)	People without sensory impairment (N = 1056)	69.1% was between 18 and 65 years old.	Women: 63,6%	NR	Hearing ratings with hearing aid if used	NR	NR	NR	Patient Health Questionnaire (PHQ-2)<br>Generalized Anxiety Disorder (GAD-2)	Multivariate logistic regression analysis	Age, gender, education, race and history of chronic conditions heart disease, hypertension, hyperlipidemia, and diabetes	No difference	No difference	Depression = All participants = AOR: 1.27 (0.69,2.23), without history op depression = AOR: 0.68 (0.20,1.76)<br>Anxiety = All participants = AOR: 1.20 (0.69,2.01), without history of anxiety = AOR: 1.20 (0.53,2.43)	Lower
Park, 2024	Depression<br>Anxiety	Prevalence	Cross-sectional	Statewide-representative research infrastructure	USA	Hearing rating fair or poor with the use of a hearing aid (N = 252)	Self-report	No hearing loss (n = 1605)	> 18 (57.1, NR))	Male: 38.7%, female: 60,3%	NR	NR	NR	NR	NR	Patient Health Questionnaire-2 (PHQ-2)<br>Generalized Anxiety Disorder-2 item (GAD-2)	Multivariable generalized linear models with a binomial distribution and a log link<br>function	Age, gender, race/ethnicity	Higher in DHH	Higher in DHH	Depression = Prevalence Ratio: 1.21 (1.06, 1.39)<br>Anxiety = Prevalence Ratio: 1.13 (1.01, 1.26)	Lower
Parker, 2021	Suicidal outcomes	Prevalence	Cross-sectional	Colleges / universities	USA	Deaf or had serious problems hearing (N = 145)	Self-report	Not deaf or serious problems hearing (N = 3067)	18 to 84 (29.7 (11))	Male: 43,4%, female: 67,6%	NR	NR	NR	NR	NR	Mini International Neuropsychiatric Interview (MINI)	Multiple logistic regression	Birth sex, race, income, age	Higher in DHH	Higher in DHH	AOR: 2.85 (1.67 to 4.87)	Lower
Parker, 2022	General mental disorders<br>Post-traumatic stress disorder<br>Substance use disorders	Prevalence	Prospective cohort	Military personal	UK	Hearing problems (N = 437), hearing problems + tinnitus (N = 377). Total N = 814	Self-report	No auditory problems (N = 3717)	18 to 66 (37.1 (NR))	Males: 87,02%, females: 12,98%	NR	NR	NR	Tinnitus presence groups	NR	General Health Questionnaire (GHQ-12)<br>National Centre for PTSD Civilian Checklist (PCL- C)<br>World Health Organization's Alcohol Use Disorders Identification Test (AUDIT)	Multivariate logistic regression analysis	Sociodemographic factors,military service factors, mental health problems (probable CMD, probable PTSD) and alcohol misuse at phase two	Mixed	No difference	General mental disorders = Hearing problems = AOR: 1.35 (0.99–1.85), Hearing problems + tinnitus = OR: 1.50 (1.09 to 2.08).<br>Post-traumatic stress disorder = Hearing problems = AOR: 0.96 (0.53 - 1.74), Hearing problems + tinnitus = AOR: 2.30 (1.41 to 3.76)<br>Substance use disorders = Hearing problems = AOR: 0.72 (0.44 to 1.17), Hearing problems + Tinnitus = AOR: 1.94 (1.28 to 2.96)	Lower
Peñacoba, 2020	Depression<br>Anxiety	Symptom severity	Cross-sectional	Community	Spain	Deaf participants, moderate (8.9%); Severe (24%); Profound (67,1%) (N = 146)	NR	Typically hearing (N = 146)	17 to 66 (DHH: 43.54 (12.5))	Male: 38,70%, female: 61,30% (179)	Being proficient in SSL (Spanish sign language) and/or SOL (Spanish oral language),	NR	NR	NR	NR	Hospital Anxiety and Depression Scale - Depression/Anxiety (HADS-D/A)	One way - MANOVA	None	Higher in DHH	Higher in DHH	Depression = DHH: 5.01 (2.69), TH: 3.27 (2.40), p < 0.001<br>Anxiety = DHH = 8.06 (3.22), TH = 6.6 (3.19), p < 0.001	Higher
Pojatić, 2019	Depression<br>Anxiety	Symptom severity	Cross-sectional	Deaf association	Croatia	Members of the Association of Deaf and Hearing-impaired Persons with hearing impairment in one ear greater than 91 decibels either congenital or acquired, lasting for at least five years. (N = 46)	NR	Without hearing impairment (N = 38)	NR (median = 43)	Men: 46,43% (39) Women: 53,57% (45)	The group of deaf people was interviewed by a researcher using the Croatian Sign Language	NR	Congenital or acquired	NR	NR	Beck Depression Inventory (BDI)<br>Beck anxiety inventory (BAI)	Kruskal Wallis H test	None	Higher in DHH	Higher in DHH	Depression = DHH median: 10 (6 - 17), TH median: 3,5 (1 - 9) P < 0.001<br>Anxiety = DHH median: 19 (13 - 26), TH median = 4 (1 - 12), p < 0.001	Higher
Reavis, 2021	Post-traumatic stress disorder	Prevalence	Cross-sectional	Military personal	USA	Participants who had normal pure-tone hearing sensitivity bilaterally, which were defined as hearing thresholds ≤ 20 dB, but with self-reported hearing difficulty (N = 100)	Self-report questionnaire	Participants who had normal pure-tone hearing sensitivity bilaterally, which were defined as hearing thresholds ≤ 20 dB, and no self-reported hearing difficulty (N = 377)	NR (31.24 (7,21))	Men: 68,9%, women: 31,1%	NR	NR	NR	NR	NR	Primary Care-PTSD- 4 screening questionnaire (PTSD-4)	Multivariable log-binomial models	Adjusted for gender, age, race/ethnicity, education, marital status, service branch, service component, service duration, combat deployment, and plus blast exposure.	Higher in DHH	Higher in DHH	PPR: 3.1 (2,2 - 4,3)	Lower
Saperstein, 2023	Psychotic disorders	Prevalence	Cross-sectional	Community	USA	Mild hearing loss (N = 25)	PTA	Participants without hearing loss (N = 140)	20 to 59 (37.9 (8.8))	Male: 59%	NR	Nobody used hearing aids	NR	NR	NR	DSM-5 diagnosis confirmed with the Mini International Neuropsychiatric Interview (MINI)	Fisher's exact test	None	Higher in DHH	Higher in DHH	OR: 4.7500 (1.6875 to 13.3701)	Higher
Schenkel, 2014	Post-traumatic stress disorder	Symptom severity	Cross-sectional	Colleges	USA	Deaf (D; n = 86), hard of hearing (HoH; n = 61) college students (67%) considered themselves to be part of the Deaf community. (Total N = 147)	Self-report	Hearing (N = 317)	NR (21.24 (4.59))	Female: 48,9%	DHH participants reported that their primary mode of communication was oral, 30 (20%) communicated using signed English, and 65 (44%) communicated using American Sign Language (ASL).	NR	NR	NR	58 (40%) went to a residential school for the deaf	The PTSD Checklist (PCL)	T-tests	None	Higher in DHH	Higher in DHH	t (462) = 3.34, p < .001.	Higher
Scinicariello, 2019	Depression	Prevalence	Cross-sectional	National representative survey	USA	High frequency or speech frequency hearing loss 26 - 40 dB, and 41dB+ (N = 1021)	Audiometric testing; air conduction thresholds	No hearing loss (≤ 25 dB). (N = 2295)	20 to 69 (41,1 (SE: 0.85))	Male: 50.6%, female: 49,4%	NR	NR	NR	NR	NR	Patient Health Questionnaire (PHQ-9)	Multivariable Logistic Regression	Age, race/ethnicity, body weight status, smoking, education, alcohol consumption, poverty income ratio, diabetes, marital status, cardiovascular disease, cancer, vision impairment, and sex	Mixed	No difference	41dB+: High frequency AOR: 1.54 (1.04–2.27), Speech frequency AOR: 1.88 (0.92–3.86); 26-40dB: High frequency AOR: 1.47 (0.98 - 2.20), Speech frequency AOR: 1.08 (0.59 to 2.00)	Lower
Shin, 2017	Depression<br>Suicidal outcomes	Prevalence	Cross-sectional	National representative survey (KHANES)	Korea	Hearing impairment<br>(N = 6395)	PTA	Non-hearing impairment (N = 12168)	20 to 59	Male: 43,32%; female: 56,68%	NR	NR	NR	NR	NR	Self-report: "Have you had depression symptoms for more than 2 weeks recently?"<br>Self-report: "Have you had suicidal ideation in the past year?"	Multivariable Logistic Regression	Adjusted with age, marital status, educational level, household income, occupation, diabetes, hypertension, body mass index, smoking, alcohol binge drinker, and physical activity.	No difference	No difference	Depression = Female 20 - 39: AOR: 0.81 (0.35–1.87); Female 40-59: AOR: 1.25 (0.89–1.75); Male 20 - 39 = AOR: 1.37 (0.54–3.46); Male 40-59 = AOR: 0.75 (0.53–1.07)<br>Suicidal outcomes = Female 20-39 years old = AOR: 1.12 (0.52–2.41), Female 40-59 years old = AOR: 1.27 (0.91–1.76), Male 20-39 years old = AOR: 1.32 (0.50–3.52), Male 40-59 years old = AOR: 0.86 (0.59–1.25)	Lower
Shiue, 2016	Depression<br>Post-traumatic stress disorder	Prevalence	Cross-sectional	Country wide population-based study	England	Ever hearing loss (N = 876)	Self-report	No ever hearing loss (N = 6165)	16 to 95 (51.1 (18.6)	Males: 43,2%, females: 56,8%	NR	NR	NR	NR	NR	Self-reported: Could you tell me if you have ever experienced any of these problems or events, at any time in your life?	Multivariate logistic regression analysis	Age, sex, body mass index (BMI), education, ethnicity, marital status, high blood pressure, smoking status and drinking status were adjusted	Higher in DHH	Higher in DHH	Depression = ever HL: AOR 1.50, 95 % CI (1.24–1.83)12-month HL AOR: 1.55 (1.25 to 1.92)<br>Post traumatic stress disorder = EVER HL: OR: 1.23 (1.03 to 1.47) 12 Month HL: OR: 1.22 (1.00–1.48)	Lower
Stam, 2016	Depression<br>Anxiety<br>General mental disorders<br>Somatic symptom disorder	Symptom severity	Prospective cohort	Community	the Netherlands	People with insufficient hearing status (57,08%), and with poor hearing status (42,92%) (N = 233)	SRTn	Good hearing status (N = 275)	18 to 69	Men: 36,6%; women: 63,4%	NR	77 (33,05%) people used a hearing aid, analyses controlled for hearing aid use	NR	NR	NR	Four- dimensional symptom questionnaire (4DSQ)	Generalized estimating equations (GEE analyses)	Gender, age, educational level, chronic health conditions, general health status, marital status, and living arrangement, Hearing aid use	No difference	No difference	Depression = β = 0.01 (−0.01 to 0.03), p = 0.198<br>Anxiety = β = 0.01 (−0.00 to 0.03) p = 0.138<br>General mental disorders = β = 0.02 (−0.00 to 0.03), p = 0.428<br>Somatic symptom disorder = β = 0.00 (−0.01 to 0.21) p = 0.573	Lower
Stefanis, 2006	Psychotic disorders	Prevalence	Prospective cohort	Schools	Greece	Hearing problems: Individuals with at least slightly bilateral hearing loss or clearly unilateral hearing loss were defined as having hearing problems. (N = 79)	Self/teacher/parent-report	This means that individuals with bilateral normal hearing or slightly unilateral hearing loss were defined as not having hearing problems. (N = 1332)	19	Male: 45,6%	NR	NR	NR	Controlling for tinnitus	NR	Community Assessment of Psychic Experiences (CAPE)	Multiple linear regression	Sex, average school grade obtained, socioeconomic status, T2 lifetime cannabis use, T2 depression, tinnitus, visual impairment at age 19, visual impairment at age 7.	Higher in DHH	Higher in DHH	b = 0.12, S.E. = 0.03, p<0.001	Lower
Suzuki, 2025	Somatic Symptom disorder<br>Sleep-wake disorders	Prevalence	Cross-sectional	National survey using stratified random sampling	Japan	Moderate to severe subjective hearing loss (N = 313)	Self-report	Without moderate to severe subjective hearing loss (N = 13745)	20-64 (Men: median age 41.0 years (IQR 29.0–52.0) without hearing loss and 53.0 years (IQR 37.0–60.0) with hearing loss; Women: 40.0 years (IQR 29.0–52.0) and 50.0 years (IQR 37.5–57.0).	Men: 47.0%, women: 53.0%	NR	NR	NR	Tinnitus in past 3 months: n = 606 (4,31% of total sample), 47,02% of DHH	NR	Somatic Symptom Scale (SSS-8)	Multiple logistic regression	Unclear	Mixed	Higher in DHH	Somatic Symptom disorder = males: OR: 3.74 (2.25 - 6.21), females: OR: 3.09 (1.69 -5.65)<br>Sleep-wake disorders = males: OR: 1.44 (0.95 - 2.18), females: OR: 1.59 (1.02 - 2.47)	Lower
Test, 2011	Sleep-wake disorders	Prevalence	Cross-sectional	National cohort	Israel	Participants exposed to harmful occupational noise during their workday, with an average decrease greater than 25 decibel A (dBA)3 in the 1000-4000 Hz range (N = 99)	PTA	Participants exposed to harmful occupational noise during their workday, with Average decrease of 25 dBA or less in the range of 1000-4000 Hz (N = 199)	NR (DHH: 52.66 (8.6))	Men: 100%	All spoken hebrew	NR	NR	51% of the DHH group, and 14,1% of the hearing group had tinnitus	NR	Mini sleep questionnaire (MSQ) - insomnia	Multivariable Logistic Regression	Years of noise exposure, tinnitus, coffee drinking, hearing impairment*tinnitus (interaction)	Higher in DHH	Higher in DHH	AOR: 3.05 (1.18 to 7.86)	Lower
Thewissen, 2005	Psychotic disorders	Prevalence	Prospective cohort	National cohort	the Netherlands	Deafness or serious hearing impairment in the past 12 months (N = 109)	Self-report	Not experienced deafness or serious hearing impairment in the past 12 months. (N = 4736)	Baseline: 18 to 64 (41.2 (11.9))	Male: 46,5%	NR	NR	NR	NR	NR	Composite International Diagnostic Interview (CIDI) - any positive response on any item	Multivariable Logistic Regression	Age, sex, education, T0 DSM-III-R psychiatric disorder, childhood trauma, experience of discrimination, urbanicity, neuroticism, cannabis use, ingle marital status, employment status, ethnic group, psychotic symptoms at T0	Higher in DHH	Higher in DHH	AOR=3.0 (1.4 to 6.2)	Lower
Tonelli, 2023	Depression<br>Psychotic disorders<br>General mental disorders	Prevalence	Retrospective cohort	Administrative health data	Canada	Hearing loss (N = 152766)	Algorithm based on Eligible ICD-9, ICD- 9-CM and ICD-10-CA	No hearing loss (N = 4571880)	>18 (DHH: 52.4 (17.5))	Male: 50.98%	NR	NR	NR	NR	NR	Validated algorithms as applied to Canadian provider claims, hospitalizations, and ambulatory care data.	negative binomial models to determine rate ratios of count out-comes by HL status<br>Odds ratio	baseline covariates, the risk of all-cause mortality, acute MI, stroke/TIA, new heart failure, new dementia and new placement, psychotic disorders and general mental disorders = none	Higher in DHH	Higher in DHH	Depression = AHR: 1.16 (1.14 to 1.17)<br>Psychotic disorders = OR: 1.8597 (1.7518 to 1.9743)<br>General mental disorders = OR: 2.41 (2.37 to 2.44)	Lower
Tremblay, 2015	Depression	Prevalence	Cross-sectional	Adult children of participants in epidemiology of hearing loss study	USA	With normal audiometric thresholds (<20dB) and self-reported hearing difficulty (N = 82)	Pure-tone air- and bone-conduction audiometry as well as word recognition in quiet and in competing message (WRCM) and self-report	With normal audiometric thresholds (<20dB) and no self-reported hearing difficulty (N = 600)	21 to 67 (DHH: 42.1 (8.4))	Male: 30,3%	NR	NR	NR	DHH: 11%, Control 5% P<0.05	NR	Center for Epidemiologic Studies Depression Scale (CES-D)	Multivariate logistic regression analysis	Age and sex	Higher in DHH	Higher in DHH	AOR: 2.39 (1.03–5.54)	Lower
van der Werf, 2011	Psychotic disorders	Prevalence	Prospective cohort	Randomly drawn sample from population registers	Germany	Childhood hearing impairment (N = 49)	Self-report and parent report using standardized checklists.	No childhood hearing impairment<br>(N = 2972)	NR (baseline: 18.3 (3.3))	Male: 49,3%	NR	NR	NR	NR	NR	Munich Composite International Diagnostic Interview (DIA-X/M-CIDI)	Multivariable Logistic Regression	Sex, education, drug abuse baseline SCL-psychosis	Mixed	Higher in DHH	AOR = 1.99 (1.06 - 3.73), 1 – 2 symptoms = ARR = 1.41 (0.65 - 3.08), 3 – 4 symptoms = ARR = 3.82 (1.31 - 11.17), 5+ symptoms = ARR = 5.66 (1.64 - 19.49)	Lower

Study, first author, year	Disorder(s)	Type of outcome	Method	Recruitment	Country	DHH group (N)	Type of audiometric test	Hearing group (N)	Age range (if reported M (SD))	Gender/sex distribution of the sample	Language used by DHH	hearing device	DHH before or after Language acquisition	Tinnitus	Education DHH	Outcome Measure	Analysis	covariates	Effect direction - Summary	Effect direction - Majority	Result / effect size / Statistic	Risk of Bias (total)
Viertiö, 2014	Psychotic disorders	Prevalence	Cross-sectional	National representative survey	Finland	Impairment in hearing (>25dB) or deaf (<br>N: WHO = 795, Conversation = 1194, Noisy environment = 2259, Interviewer = 759	Screening audiometer, self-report, interviewer rating	No hearing impairment (<25 dB)<br>N: WHO = 6044, Conversation = 5645, Noisy environment= 4580, Interviewer = 6080	>30	NR	NR	Participants used their hearing aids during the interview	NR	NR	NR	SCID-I/medical records	Odds ratio	none	Mixed	No difference	Schizophrenia =<br>WHO = OR: 1.2660 (0.5998 to 2.6724), Hearing conversation = OR: 2.1429 (1.2202 to 3.7633), difficulties hearing in a noisy environment = OR: 1.9750 (1.1925 to 3.2710), Interviewer rating = OR: 1.2114 (0.5738 to 2.5575)<br>other non-affective psychotic disorder =<br>WHO= OR: 0.9772 (0.4862 to 1.9640), Hearing conversation = 1.1214 (0.6603 to 1.9044), difficulties hearing in a noisy environment = OR: 1.3186, (0.8371 to 2.0772), Interviewer rating = OR: 0.6557 (0.2842 to 1.5125),<br>Affective psychosis =<br>WHO= OR: 1.1709 (0.4941 to 2.7746), Hearing conversation = 2.1486 (1.1395 to 4.0514), difficulties hearing in a noisy environment = OR: 1.9485 (1.0837 to 3.5035), Interviewer rating = OR: 1.2342 (0.5208 to 2.9250)	Lower
Wallace, 2020	Depression<br>Anxiety	Prevalence	Cross-sectional	Population-based sampling regional / district	Cameroon, Guatemala, India, Maldives, Nepal	Functional difficulty hearing (even when using hearing aids) (Cameroon, N = 32; Guatemala, N = 120; India, N = 85; Maldives, N = 36; Nepal, N = 48))	Self-report	People without functional hearing difficulties or some difficulty (Cameroon, N = 1567; Guatemala, N = 7481; India, N = 2247; Maldives, N = 3364; Nepal, N = 3934))	Cameroon = 18 to 99 (44.5 (NR)); Guatemala = 18 to 100 (39.2 (NR)), India = 18 to 98 (39.2 (NR)), Maldives = 18 to 102 (39.3 (NR)), Nepal = 18 to 96 (43 (NR)	% female Cameroon = 69,8%, Guatemala = 57%, India = 54,3%, Maldives = 57.5%, Nepal = 57.7%	NR	Cameroon = 2 (0.1%) used hearing aids , Guatemala = 27 (0.4%) used hearing aids , India = 3 (0.1%) used hearing aids, Maldives = 23 (0.6%) used hearing aids, Nepal = 11 (0.3%) used hearing aids	NR	NR	NR	Relevant Washington Group Extended Question Set on Functioning Questions	Multivariate logistic regression analysis	Age and sex	Mixed	No difference	**Depression**<br>Cameroon = Moderate depression = AOR: 0.8 (0.3–2.2), severe depression = AOR: 0.9 (0.2, 4.2), Guatemala = Moderate depression = AOR: 1.3 (0.7, 2.2), Severe depression = AOR: 6.3 (3.8, 10.4), India = Moderate depression = AOR: 1.0 (0.6, 1.8), severe depression = AOR: 1.9 (0.9, 3.8), maldives = Moderate depression = AOR: 1.7 (0.4–7.5), severe depression = AOR: 2.1 (0.8–5.7), Nepal = Moderate depression = AOR: 4.9 (2.3, 10.2), severe depression = AOR: 11.7 (3.8, 36.5)<br>**Anxiety**<br>Cameroon = Moderate anxiety = AOR: 1.1 (0.5, 2.5), severe anxiety = AOR: 1.4 (0.4, 5.0), Guatemala = Moderate anxiety = AOR: 1.5 (0.9, 2.3), severe anxiety = AOR: 4.9 (3.1, 7.8), India = Moderate anxiety = AOR: 1.0 (0.6, 1.7), severe anxiety = AOR: 1.7 (0.9, 3.2), Maldives = Moderate anxiety = AOR: 0.5 (0.1–4.2), severe anxiety = AOR: 2.8 (1.2–6.5), Nepal = Moderate anxiety = AOR: 2.4 (0.9, 6.2), severe anxiety = AOR: 9.6 (3.5, 26.6).	Lower

Supplement G

Risk of bias of studies included in the systematic review

Study<br>First author, year *(outcome)*	Domain 1 Represent-ativeness	Domain 2 Match exposed non-exposed	Domain 3 Assessment of exposure	Domain 4 Assessment of the outcome	Domain 5 Confoun-ding	Domain 6 Assessment of confounding	Domain 7 Missing data	Number of Low risk domains
Ahrenfeldt, 2024	Low	Low	High	Low	Low	Low	High	(5/7)
Akobirshoev, 2022 *(SUD-opiod)*	High	Low	Low	Low	Low	Low	Low	(6/7)
Akobirshoev, 2022 (*all other outcomes)*	High	Low	Low	Low	High	High	Low	(4/7)
Akram, 2020	High	Low	High	Low	High	High	unclear	(2/7)
Aram, 024	Low	Low	High	Low	Low	Low	Low	(6/7)
Awad, 2024	Low	Low	Low	High	Low	Low	unclear	(5/7)
Bakir, 2013	High	Low	Low	Low	High	High	unclear	(3/7)
Bernard, 2022	High	Low	Low	Low	Low	Low	High	(5/7)
Bigelow, 2020 *(depression and anxiety)*	Low	Low	High	High	Low	Low	unclear	(4/7)
Bigelow, 2020 *(general distress)*	Low	Low	High	Low	Low	Low	unclear	(5/7)
Bikbov, 2023 *(suicide)*	Low	Low	Low	High	Low	Low	low	(6/7)
Bikbov, 2023 *(all other outcomes)*	Low	Low	Low	Low	Low	Low	low	(7/7)
Bizjak, 2009	High	High	High	Low	High	High	unclear	(1/7)
Choi, 2021	Low	Low	Low	Low	Low	Low	Low	(7/7)
Ciesla, 2016	High	unclear	Low	Low	High	High	unclear	(2/7)
Cormier, 2024	High	Low	High	Low	Low	unclear	High	(3/7)
Cui, 2025	Low	Low	High	High	Low	Low	High	(4/7)

Dammeyer, 2017	Low	unclear	High	High	High	High	high	(1/7)
Del Vecchio, 2023	High	Low	Low	Low	High	High	unclear	(3/7)
Fellinger, 2005	High	High	High	Low	High	High	unclear	(1/7)
Fors, 2013	High	Low	Low	Low	Low	Low	High	(5/7)
Fox, 2020	High	Low	High	High	Low	Low	High	(3/7)
Gao, 2022	Low	Low	High	Low	Low	Low	High	(5/7)
Genc, 2013	Unclear	unclear	Low	Low	Low	Low	unclear	(4/7)
Grewal, 2023	High	Low	Low	Low	Low	Low	High	(5/7)
Hou, 2025	High	Low	Low	Low	High	High	unclear	(3/7)
Hsu, 2016	Low	Low	Low	Low	Low	Low	Low	(7/7)
Idstad, 2019	High	unclear	Low	Low	Low	Low	unclear	(4/7)
Keidser, 2015	Low	Low	Low	High	High	Low	unclear	(4/7)
Kushalnagar, 2017	High	Low	High	Low	High	Low	unclear	(3/7)
Kushalnagar, 2019	High	Low	High	High	High	Low	unclear	(2/7)
Lee, 1997	High	Low	Low	Low	Low	Low	Low	(6/7)
Leigh, 1989	High	unclear	Low	Low	High	Low	unclear	(3/7)
Li, 2014 *(depression)*	Low	Low	High	Low	Low	Low	unclear	(5/7)
Li, 2014 *(sleep)*	Low	Low	High	unclear	High	High	unclear	(2/7)
Linszen, 2019	High	Low	Low	High	Low	unclear	Low	(4/7)
MacGregor, 2023	High	Low	High	Low	Low	Low	unclear	(4/7)
Marlow, 2021	Low	Low	High	High	Low	Low	unclear	(4/7)
McClintock, 2023	Low	Low	High	Low	Low	Low	unclear	(5/7)
McDermott, 2005	High	Low	Low	Low	High	High	Low	(4/7)
McKee, 2019	Low	Low	High	High	Low	Low	unclear	(4/7)
Meher, 2022	Low	Low	High	Low	Low	Low	unclear	(5/7)

Merten, 2023	Low	Low	High	Low	Low	Low	High	(5/7)
Park, 2024	Low	Low	High	Low	Low	Low	unclear	(5/7)
Parker, 2021	High	Low	High	Low	Low	Low	High	(4/7)
Parker, 2022	High	Low	High	Low	Low	Low	Low	(5/7)
Peñacoba, 2020	High	Low	Low	Low	High	High	unclear	(3/7)
Pojatić, 2019	High	unclear	Low	Low	High	High	unclear	(2/7)
Reavis, et al., 2021	High	Low	Low	Low	Low	Low	unclear	(6/7)
Saperstein, 2023	High	Low	Low	Low	High	High	unclear	(3/7)
Schenkel, 2014	High	Low	High	Low	High	High	unclear	(2/7)
Scinicariello, 2019	Low	Low	Low	Low	Low	Low	Low	(7/7)
Shin, 2017	Low	Low	Low	High	Low	Low	unclear	(5/7)
Shiue, 2016	Low	Low	High	High	Low	Low	unclear	(4/7)
Stam, 2016	High	Low	Low	Low	Low	Low	unclear	(5/7)
Stefanis, 2006	Low	Low	High	Low	Low	Low	unclear	(5/7)
Suzuki, et al., 2025	Low	Low	High	Low	High	Low	High	(4/7)
Test, 2011	High	Low	Low	Low	High	Low	unclear	(4/7)
Thewissen, 2005	Low	Low	High	Low	Low	Low	unclear	(5/7)
Tonelli, 2023 *(general and schizophrenia)*	Low	Low	Low	Low	High	High	unclear	(4/7)
Tonelli, 2023 *(depression)*	Low	Low	Low	Low	Low	Low	unclear	(6/7)
Tremblay, 2015	High	Low	Low	Low	High	Low	unclear	(4/7)
van der Werf, 2011	Low	Low	High	Low	Low	unclear	unclear	(4/7)
Viertio, 2014	Low	Low	Low	Low	High	High	High	(4/7)
Wallace, 2020	Low	Low	High	High	High	Low	Low	(4/7)
